# Optoelectronic Nanofluidic Neural Networks for Ionic Computing

**DOI:** 10.1002/adma.74080

**Published:** 2026-07-16

**Authors:** Yaxin Huang, Zhiyuan Du, Changjin Wu, Binglin Zeng, Yingnan Cao, Mingliang Li, Liren Zhang, Qingxin Guo, Chao Huang, Can Li, Jinyao Tang

**Affiliations:** ^1^ School of Mechanical Engineering Beijing Institute of Technology Beijing P. R. China; ^2^ Department of Chemistry The University of Hong Kong Hong Kong P. R. China; ^3^ Department of Electrical and Electronic Engineering The University of Hong Kong Hong Kong P. R. China; ^4^ State Key Laboratory of Synthetic Chemistry The University of Hong Kong Hong Kong P. R. China; ^5^ HKU‐CAS Joint Laboratory on New Materials and Department of Chemistry Hong Kong P. R. China; ^6^ Materials Innovation Institute for Life Sciences and Energy (MILES) The University of Hong Kong Hong Kong P. R. China

**Keywords:** fluidic memristor, in‐sensor computing, ionic memory, reservoir computing

## Abstract

Emerging neuromorphic computing technologies aim to replicate the brain's cognitive processes and offer promising capabilities for advanced computation and complex tasks. Building artificial neural networks (ANNs) with robust connections and dynamic plasticity is essential for their implementation. Nevertheless, most existing ANNs rely on solid‐state semiconductors, where electrons or holes serve as information carriers. Here, we present a novel ion‐based nanofluidic memristor operating in aqueous solutions, which can be integrated to create densely connected ionic neural networks (INNs). The nanofluidic memristor exhibits tunable ionic memory and diverse synaptic plasticity that can be modulated by electrical, optical, or combination signals. We demonstrate that the ionic memory originates from non‐trivial hysteretic ion‐charged surface interactions in confined nanochannels, supported by experiments and numerical calculations. Utilizing the tunable states of the nanofluidic memristor, we implement reservoir computing to classify static patterns and dynamical motions, achieving ultrahigh accuracies of 91% and 97% on the standard MNIST datasets and homemade moving‐particle library, respectively. Multiple nanofluidic memristors are integrated to construct a practical INN capable of real‐time logic computation and in‐sensor computing, verifying the robustness of nanofluidic computation systems. This ion‐based nanofluidic platform offers a promising pathway towards fully connected INNs and advances neuromorphic computing beyond solid‐state electronics.

## Graphical abstract

An ion‐based optoelectronic nanofluidic memristor enables neuromorphic computing in aqueous environments. With tunable ionic memory and multimodal synaptic plasticity, it realizes densely connected ionic neural networks capable of image classification, motion prediction, logic computation, and real‐time in‐sensor computing, advancing fully connected neuromorphic architectures beyond solid‐state electronics.

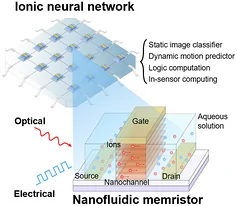

## 1 Introduction

Neuromorphic computing has advanced significantly in recent years, with a focus on replicating the complex, energy‐efficient information‐processing capabilities of the human brain [1]. Current neuromorphic computing technologies predominantly rely on solid‐state resistance‐switchable devices, such as memristors or field‐effect transistors [2], which regulate the transport of electrons or holes in semiconductors. However, biological neurons operate by modulating ion transport in a highly ion‐conductive aqueous solution, facilitating complex functions with higher efficiency [3]. While solid‐state devices like memristors [4, 5] have been developed to display diverse synaptic plasticity [6, 7], their structural incompatibility with biological systems presents a substantial challenge. Thus, a fluidic ionic device that can emulate neuro‐fluidic behaviors, with potential applications in neuromorphic computing, bioelectronics, and beyond, is highly desirable.

The rapid advancement in nanofluidics has led to the discovery of unique ion transport phenomena in spatially confined environments, such as ultrafast transport [8, 9], and extreme selectivity [10]. This has facilitated the development of advanced ionic devices like ionic sensors [11], diodes [12], and transistors [13], which are crucial for ionic integrated circuits. Recent theoretical predictions [14] and experimental investigations [15, 16] have demonstrated the formation of ionic memory in fluidic memristors, which display a voltage history‐dependent ionic conductivity in nanoscale pores or micro‐pipettes in aqueous electrolytes. These fluidic systems exhibit unique ion‐channel interactions, such as ionic self‐assembly [15], surface adsorption [16], or reversible formation of liquid blisters [17], which play significant roles in the nonlinear transport for the formation of ionic memory, allowing for a new asset to fine‐tune the properties of conductive electrolytes, oppositely to their bulk counterparts. However, the implementation of fluidic memristors at the circuit scale has been considerably limited by the poor mechanical stability of ultrathin membranes [15, 17], the intricate fabrication process of extremely narrow channels [15, 16, 18, 19], and unimodal synaptic plasticity [20, 21]. While biological systems rely on collaborative interactions among multiple neurons with densely interconnected synapses, current fluidic memristors, typically consisting of a single or a few units [16, 17, 22], fall short in performing complex computations like pattern classification [23] and motion recognition [24]. Therefore, overcoming these challenges in fabrication and integration is crucial to achieving scalable, uniform, and multimodal fluidic memristors for building INNs and implementing complex computation tasks.

This work presents an optoelectronic nanofluidic memristor (OENFM) that synergistically combines nanoconfined ionic transport with photoresponsive modulations to enable dynamic and tunable control of ionic conductance states. This unique functionality allows the OENFM to be effectively integrated into INNs for implementing diverse and multi‐task computations. The OENFM exhibits a pitched ionic hysteresis in aqueous electrolytes, indicative of the formation of electrical history‐dependent ionic memory. The memristor demonstrates multimodal responsiveness modulated by optical, electrical, or hybrid signals. Various chemical features of embedded solutions, including electrolyte concentration, pH value, and ionic types, provide versatile measures for memory regulation. The OENFMs also display a wide range of plasticity, including short‐term and long‐term potentiation/depression (STP/STD, LTP/LTD), as well as pair‐pulse facilitation/depression (PPF/PPD), allowing for the construction of INNs for different computational purposes. We exploit the optical response of the OENFM to build a physical reservoir with 32 nodes, achieving over 91% and 97% accuracy in image classification from the MNIST dataset and dynamical motion recognition with a homemade moving particle library, respectively. More importantly, the OENFMs can be effectively scaled and integrated with CMOS‐compatible microfabrication methods, facilitating circuit‐scale ionic computing. As a proof‐of‐concept, we demonstrate the successful integration of a 3 × 3 × 3 memristor array that functions as a sensitive image sensor, enabling in‐sensor ionic computing, where three different images can be accurately distinguished. This work offers a practical approach for constructing fluidic memristor‐based integrated circuits and systems, paving the way for implementing advanced ionic computing in aqueous environments.

## 2 Results and Discussion

### 2.1 Ionic Neural Networks Based on Optoelectronic Nanofluidic Memristors

**FIGURE 1:**
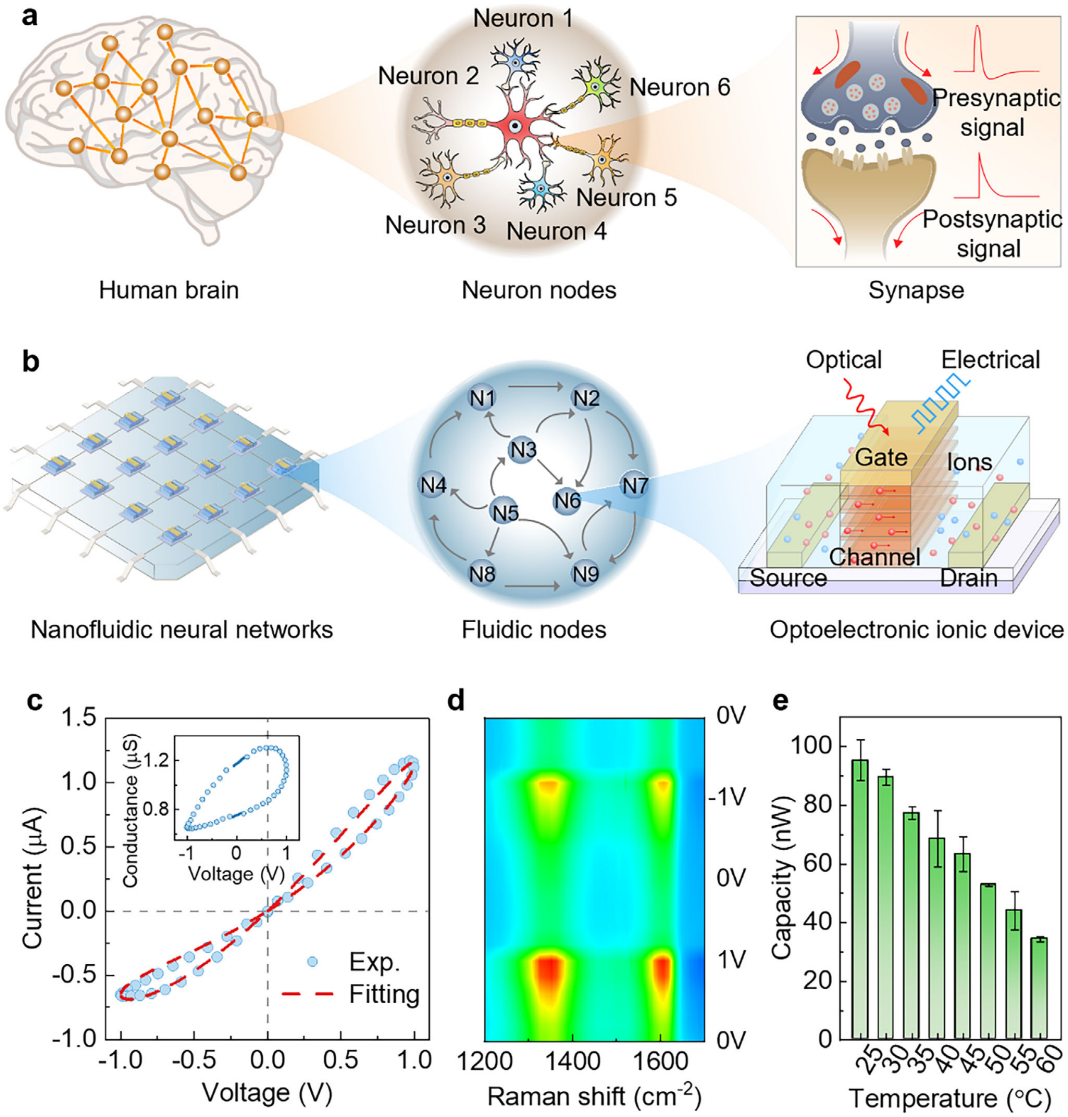
Bioinspired nanofluidic‐based ionic neural networks (INNs). (a) Graphical representation of the human brain, illustrating the intricate network of neurons and synapses. As the core structural unit, the synapse connects different neurons and plays a crucial role in stimuli perception, signal processing, and decision‐making in the brain. When exceeding a specific threshold, the external stimuli activate the synapse, leading to a notable post‐synaptic signal. The dynamic synaptic connection, characterised by varying weights, determines the computation process and operation implementation. (b) Schematic illustrations of bioinspired nanofluidic‐based ionic neural networks, consisting of an array of fluidic nodes based on optoelectronic nanofluidic memristors (OENFMs). The ionic transport in these memristors can be easily modulated by imposing optical, electrical, or optoelectronic fields. The resulting adjustable ionic conductance, analog to the synaptic weights, allows dynamic control over the formed INNs, facilitating different computation. (c) Current‐voltage curve of the OENFM measured in an aqueous KCl solution, depicting a typical voltage‐history‐dependent hysteresis. The inset showcases the calculated conductance‐voltage curve. (d) Raman mapping spectrum of the PNCs at different scanning voltages, exhibiting a potential‐dependent hysteretic variation. The red and blue colors indicate higher and lower strength, respectively. (e) Hysteretic loop area as a function of testing temperatures, which significantly decreases with increased temperature. All tests were conducted in 1 mM KCl solutions (pH ≈ 6.8).

The realization of advanced functionalities in the human brain relies on the dynamic change of neuron networks, where the synapses, as crucial components, release neurotransmitters in response to presynaptic stimuli activation. Synaptic weights adapt according to the intensity and spatiotemporal patterns of stimuli, enabling efficient information transmission and logical processing (Figure 1a). Inspired by this biological process, we propose the design of ionic neural networks (INNs) based on OENFMs that can be modulated by optical or electrical signals (Figure 1b). The unique ion transport within the confined nanochannels of OENFM enables the formation of an intricate hysteretic ionic memory, and the tunable multi‐plasticity further facilitates ionic computations, representing a promising way for creating an ionic chip.

The OENFM was built on highly ordered photoresponsive nanochannels (PNCs) on a silicon substrate, consisting of two‐dimensional graphene oxide nanosheets decorated with photoresponsive molecules (pyranine) through non‐covalent interactions in an ordered way via spin‐coating method (Figure S1). As shown in Figure S2, XRD analysis shows a diffraction peak at 2θ = 11.3° for the PNCs, compared to 11.9° for pristine graphene oxide nanochannels (NC), indicating an expanded interlayer spacing after pyranine incorporation. Raman spectra reveal no appreciable shifts in the D or G bands, implying minimal structural disruption. EDX analysis detects 6.0 at% sulfur in the PNCs, whereas sulfur is absent in pristine NCs, confirming successful pyranine loading. FT‐IR spectra of PNCs exhibit a characteristic pyranine peak [25] at 1180 cm^−^^1^, which is not observed in pristine NCs. Photolithography microfabrication was employed to define the channel geometry and create fluidic reservoirs for executing aqueous electrolyte measurements (See details in Supporting Methods). The resulting PNCs consist of multilayer nanochannels, which exhibit a typical transport length of 50 µm, an effective width of 2 mm, and a thickness of approximately 50 nm, as defined by the microfabrication process. The average interlayer spacing is estimated to be approximately 0.8 nm from the XRD patterns (Figures S1 and S2). The measured ionic conductance increases linearly with the PNC thickness, consistent with the well‐defined lamellar spacing and the continuous, relatively smooth morphology (Figure S1). With such ultra‐confinement, ions and water molecules experience nearly single‐digit transport [26], leading to ion accumulation or depletion when co‐ions or counter‐ions pass through the PNCs. This inherent nonlinear ionic transport is crucial for forming ionic memory in a fluidic context. The horizontal nanochannel configurations and miniaturized reservoirs, facilitated by the scalable and CMOS‐compatible microfabrication process, allow for batch‐level fabrication and integration of OENFMs. This accomplishment marks a significant advancement over previous methods for building INNs for executing neuromorphic computing.

We first measured the ionic transport through PNCs using a pair of redox electrodes (Ag/AgCl) to detect the ionic current, with the clamping reservoirs filled with aqueous potassium chloride solution (1 mM – 1 M). When a sinusoidal electric potential is applied, the ionic current of PNCs exhibits a distinct pitched loop (Figure 1c), a typical characteristic of a memristor [4, 27]. We plotted the conductance curve against the applied electric bias, which increases at positive voltages and decreases at negative ones, demonstrating significant memory retention at low voltages, indicative of a bipolar memristor [15]. The hysteretic current‐voltage curve is further fitted according to a minimal model given by the convolution of quasistatic conductance with an exponential memory kernel [15].

$$G\left(t\right)=\int_{0}^{\infty }G_{qs}\left[V\left(t-s\right)\right]\frac{e^{-s/\tau }}{\tau }\;ds$$

where G_qs_ is quasistatic (nonlinear) conductance, V(t) is the applied voltage, and τ is a time scale order of memory duration. The experimental results closely align with the theoretical predictions (Figure 1c). According to the model, we can extract the memory time of the PNCs τ to be approximately 150 seconds, considerably longer than previously reported micropores [16, 18, 28] and comparable to the ultra‐confined nanometric slits [15, 17]. This extended memory is further confirmed by prolonged conductance retention after applying a transient positive spike below the water electrolysis potential (Figure S3) [22, 29]. Generally, the diffusion time in the PNCs (L ≈50 µm in length) can be roughly estimated by L^2^/4D, where D is the ionic diffusion coefficient of mobile cations (K^+^, 1.96 × 10^−9^ m^2^/s). The calculated diffusion time is about 1.25 s, two orders of magnitude smaller than the observed memory time (150 s) in the PNCs, suggesting that interfacial processes, such as ion adsorption [30], rather than diffusion, govern the nonlinear ion transport and determine the formation of ionic memory in the PNCs. Alternating positive and negative voltage pulses were applied to program the PNCs, which exhibited reversible switching between high‐ and low‐conductance states over repeated cycles, confirming their set/reset switching capability [20] (Figure S3).

To investigate ion adsorption on the PNCs, we conducted in situ spectroelectrochemical Raman measurements [31, 32]. These spectra revealed broad bands at 1370 cm^−1^ and 1620 cm^−1^, characteristic features of D and G bands of the graphene oxide, respectively (Figure S4). We observed negligible peak shifts at varied electric bias applied on the PNCs, and only relative intensity variations with a constant calculated I_D_/I_G_ ratio (1.04–1.07), suggesting no chemical reactions occurred in the PNCs during electrical scanning. Notably, the characteristic D and G band intensities gradually increased upon increasing the applied potential from 0 V to 1.0 V, and subsequently returned to the initial value at 0 V; when the applied potential became negative (−1.0 V), the intensity correspondingly intensified, and a maximum peak intensity (indicated by red color) was observed at a potential of −0.7 V, showcasing a significant intensity hysteresis (Figure 1d). This hysteresis suggests that ion migration does not immediately equilibrate with the applied potential due to ionic memory. Specifically, ions driven toward the surface at −1.0 V persist as the potential reverses, shifting the Raman signature of adsorbed ions to a maximum at −0.7 V. In the absence of such memory or with faster ion equilibration, the Raman peak would align with the potential at −1.0 V. These results demonstrate that ion adsorption dynamics lag behind the applied potential, producing voltage‐history‐dependent Raman signatures characteristic of ion adsorption on PNCs. Control experiments using pristine GO nanochannels show similar voltage‐dependent memristive behavior but with reduced hysteretic area (Figure S5). Given the thermodynamic nature of ion adsorption, we hypothesized that temperature would significantly affect the process and provide insights into the ionic conductivity hysteresis mechanism. The ion hysteresis effect, also known as ionic memory capacity, can be quantified based on the area enclosed by the current‐potential curves. As shown in Figure 1e, the hysteresis loop area gradually decreases with increasing temperature in the range from room temperature to 60°C. This temperature‐induced reduction arises from the combined effects of weakened interfacial ion adsorption and accelerated ion transport. Elevated temperature shortens residence time of ions on the nanochannels and is known to decrease electrolyte viscosity [33] and increase electrolyte conductivity [34], thereby accelerating ion redistribution in the confined nanochannels. Consistently, the measured ionic conductance of the PNCs exhibits Arrhenius‐type temperature dependence [35], with an apparent activation energy of approximately 0.188 eV (Figure S5), directly demonstrating thermally activated ion transport. As a result, the interfacial ionic state follows the applied voltage more rapidly, responsible for ionic hysteresis and leading to smaller memory capacity.

To further examine the voltage‐dependent ionic hysteresis in the PNCs, we performed electrochemical quartz crystal microbalance (EQCM) measurements. As shown in Figure S6, under a constant applied potential, the PNC film exhibits a time‐dependent frequency shift that gradually approaches a plateau over several hundred seconds, comparable to the characteristic memory timescale of the device. Moreover, the magnitude of the frequency response decreases with increasing temperature, consistent with accelerated ion transport and adsorption/desorption kinetics, which reduce the retention of ions and solvent within the confined interlayer nanochannels. This temperature dependence agrees with the observed decrease in memory capacity at elevated temperatures (Figure 1e). During cyclic voltage scanning, the frequency response exhibits a clear history‐dependent variation. Notably, for hydrated PNCs, the EQCM response may reflect not only ion adsorption/desorption but also hydration‐water redistribution and changes in viscoelastic coupling [36], which may contribute to the observed positive frequency changes [37, 38]. Therefore, the EQCM results provide complementary evidence for the involvement of delayed interfacial ionic processes in the memory behavior of the PNCs. The delayed Raman response during potential sweeps, the temperature‐dependent reduction in memory capacity, and the EQCM frequency response collectively provide comprehensive evidence that coupled ion adsorption and ion‐transport kinetics underline the observed ionic hysteresis. These findings are consistent with interfacial ion adsorption thermodynamics [39], where increased temperature provides additional kinetic energy for adsorbed ions to escape from trapped states to isolated states, mitigating ion adsorption and resulting in a weakened memory capacity. These results underscore the significant role of surface ion adsorption in the formation of ionic memory in the PNCs.

### 2.2 Electrical Synaptic Plasticity of Nanofluidic Channels

**FIGURE 2:**
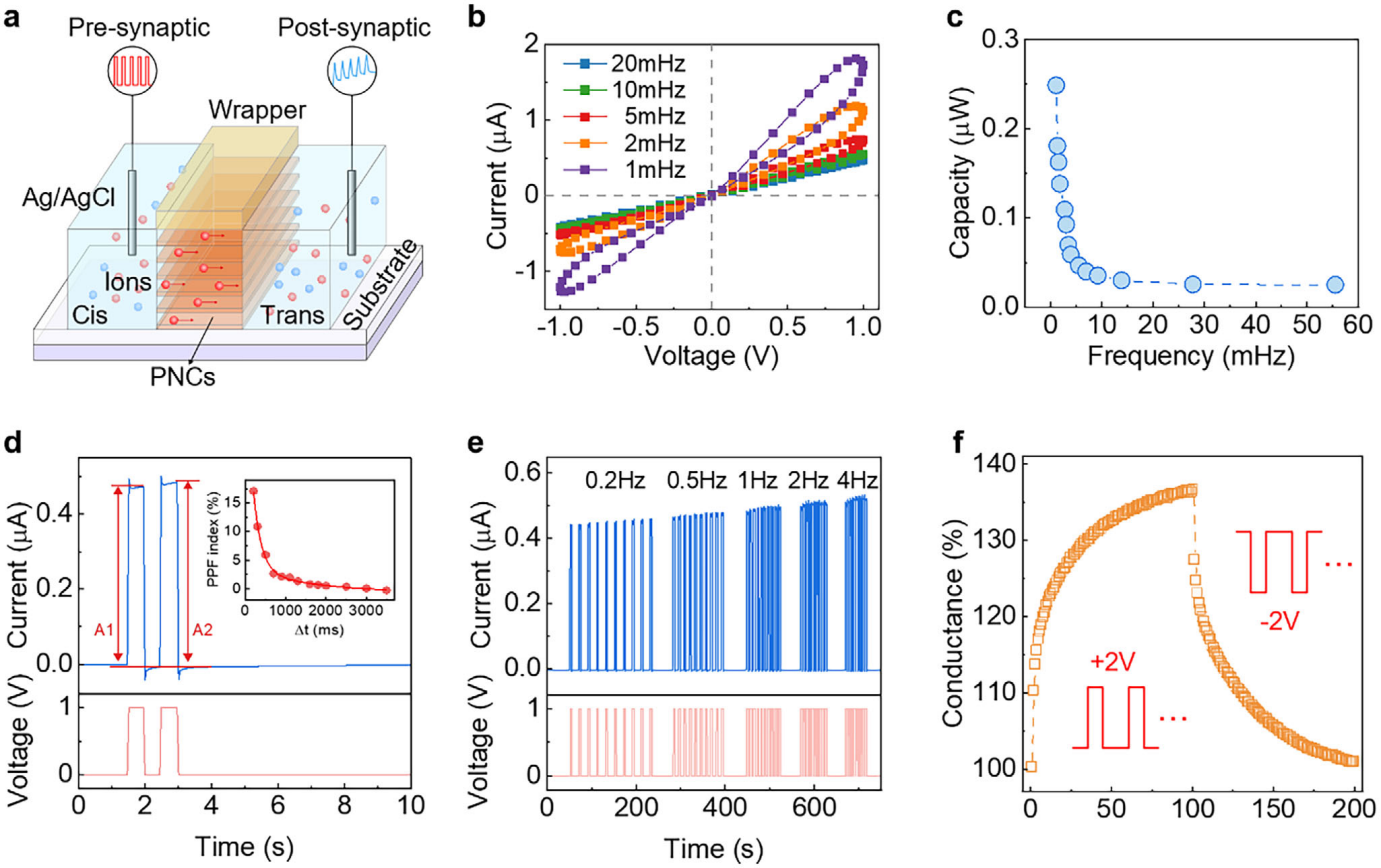
Electrical plasticity of the OENFM. (a) Schematic of the electrical modulated synaptic plasticity measurements on the OENFMs. The applied electric fields function as the presynaptic signal, and the ionic transport through the PNCs is recorded as the post‐synaptic signal using a pair of redox electrodes. Altering the imposed electrical pattern allows for the examination of the electrical plasticity of the OENFMs. (b) Current‐voltage curves of the OENFMs under varying scanning frequencies demonstrate an enhanced hysteresis at lower frequencies. (c) Memory capacity dependence on the scanning frequency, rapidly diminishes with increased scanning frequencies and approaches zero when the frequency exceeds 20 mHz. (d) Paired‐pulsed electrical stimulation on the OENFMs by utilizing a pair of pulsed voltages (1 V, 0.5 s, with an interval of 0.5 s) to activate the device. The responsive post‐synaptic current exhibits a pair‐pulsed facilitation (PPF) effect, with the amplitude ratio (A_2_–A_1_)/A_1_ defined as the PPF index. The inset depicts the PPF index trend on the pulse intervals under fixed pulse voltages and duration (1 V, 0.5 s). (e) Sequential pulsed electrical stimulation and the current response at varied frequencies. A higher stimulation frequency leads to a pronounced post‐synaptic current. (f) Long‐term potentiation with 100 positive pulses (2 V, 100 ms) and long‐term depression with 100 negative pulses (−2 V, 100 ms), with the conductance state read at a lower bias (0.05 V). All tests were conducted in 1 mM KCl solutions (pH∼6.8).

The electrical response of the PNCs is systematically investigated by applying diverse electric signals (Figure 2a). As shown in Figure 2b, the PNCs exhibit an enhanced ionic hysteresis at lower scanning frequencies upon sinusoidal stimulation. When the frequency exceeds 20 mHz, the memory capacity approaches nearly zero, suggesting a long relaxation time (on minute scale) for ions interacting with the PNC's charged surface (Figure 2c). This frequency‐dependent memory is found to be waveform‐independent (Figure S7). At frequencies above 20 mHz, hysteresis becomes negligible due to the inability of mobile ions in the PNCs to follow the rapid external potential changes, consistent with previous research [15]. As such, hysteresis studies are generally implemented within the 1–20 mHz frequency range. Specifically, these PNCs display a slight ion rectifying effect at varied KCl concentrations (0.1–10 mM), with rectification ratios of approximately 1–1.03 (Figure S8). This weak rectification is consistent with the uniform channel structure as revealed by the cross‐sectional SEM images (Figure S1), which substantially contrasts with previous studies on asymmetric nanopores (9.5–14) [40] or ultra‐narrow nanochannels (1–5) [15]. Minor rectification arises from the multilayered structure with uniform channel height and eliminates the effects of electrolyte concentration polarization at the entrances or exits [41].

The electrical plasticity of the PNCs is also closely associated with chemical environments, such as electrolyte concentrations, cation types, and pH conditions (Figure S9). Increasing electrolyte concentration reduces the memory capacity, consistent with stronger electrical double layer screening. For monovalent cations, the normalized hysteretic memory capacity of the PNCs follows the sequence of K^+^> Na^+^ > Li^+^, aligning with the mobility order µ_K+_> µ_N__a+_ > µ_L__i+_ [42]. For multivalent cations, such as Mg^2+^ and Al^3+^, Al^3+^ exhibits a larger normalized memory capacity than Mg^2+^, indicating that the ion‐specific memory behavior also involves coupled contributions from Coulombic adsorption, dehydration, and others [30]. Moreover, the normalized memory capacity increases under basic conditions and nearly vanishes at lower pH values (i.e., pH = 2). This can be interpreted by the tunable ion‐sieving capability of the PNCs, which display increased surface charge density under basic conditions and reduced surface charge density under acidic conditions, corroborating the notable role of surface charges in ionic memory formation. In addition, we implemented cyclic conductance‐voltage measurements of the PNCs. The normalized memory capacity shows no obvious degradation during repeated operation (Figure S9). This repeatability is attributed to the reversible ion adsorption/desorption dynamics at the PNC interfaces. These tunable parameters, akin to biological ion channels, are unique to nanofluidic systems and challenging to achieve in solid‐state electronic devices that rely exclusively on electron/hole flow.

To investigate the temporal electrical dynamics, transient voltage spikes, including single, paired, and cyclic‐pulsed signals are applied to the PNCs (Figures S10 and S11). The post‐synaptic current (PSC) exhibits a rapid increase when a positive voltage pulse is applied, followed by a swift reversal and gradual decay to its initial state, and it intensifies with higher potentials. A similar trend, but with opposite values, is observed with a negative pulse. The pair‐pulsed plasticity of the PNCs is examined by applying pairs of positive or negative pulses at varied intervals. If the second pulse arrives before the PSC from the first pulse fully decays, an accumulative effect results in a higher or lower PSC spike, indicating spike‐rate‐dependent plasticity. Shorter pulse intervals (Δt) lead to increased current change (ΔI), while longer intervals result in a decrease. As shown in Figure 2d, the current change under positive pulses exhibits a pair‐pulsed facilitation (PPF) behavior and follows a biexponential relationship with the pulse interval:

$$\Delta I=A_{0}+A_{1}e^{-\frac{\Delta t}{\tau_{1}}}+A_{2}e^{-\frac{\Delta t}{\tau_{2}}}$$

where τ_1_ and τ_2_ represent time constants for ion interactions in the reservoir and PNCs, respectively, and A_0_, A_1_, and A_2_ are factors weighting these dynamic processes. Fitting the current change against the pulse interval curve yields time constants τ_1_ = 0.192 s and τ_2_ = 1.53 s, which are comparable to biological systems and previous research [16]. In contrast, negative pulses result in a gradual current decrease with extended pulse intervals, suggesting notable pair‐pulse depression (PPD) behavior (Figure S11). The polarity‐dependent paired‐pulse behavior arises from cation accumulation or depletion near the negatively charged PNC surface. Positive pulses promote interfacial cation accumulation and residual ion occupancy before the second pulse, leading to PPF, whereas negative pulses deplete interfacial cations and further suppress the second response, resulting in PPD. By applying a train of positive pulses with increasing frequency (0.2, 0.5, 1, 2, and 4 Hz) to the PNCs, there is a prominent increase in both maximum and minimum PSC values (Figure 2e), indicating that the conductance does not fully relax to its initial state between adjacent pulses. Repeated stimulation leads to cumulative ion accumulation and/or adsorption within the confined nanochannels, leaving a residual interfacial ionic state after each pulse. In contrast, highly reproducible current responses are observed at lower stimulation frequency of 0.1 Hz (Figure S11), suggesting that longer pulse interval permits sufficient ionic relaxation. At higher pulse frequencies, the shorter relaxation interval enhances this cumulative effect, resulting in stronger conductance potentiation. This pulse‐number‐ and frequency‐dependent response reflects history‐dependent ionic memory in the PNCs and provides the basis for synaptic plasticity. Furthermore, the PNCs demonstrate long‐term conductance modulation capabilities, including both long‐term potentiation (LTP) and long‐term depression (LTD), by applying repeated pulsed signals of different polarities (Figure 2f).

The PNC's electrical response, characterized by apparent inversed signals that intensify with higher pulse voltages observed at the initiation and cessation of applied pulsed voltage, significantly differs from previous solid‐state electronic memristors [23, 43] and has not been documented in ultra‐confined nanochannels below a few nanometers [15, 17]. As recently suggested by Juan et al. [44, 45], a chemical inductor might be responsive to this unique inductive behavior and contribute to ionic memory formation in fluidic systems. To verify the presence of this chemical inductive behavior in our nanofluidic device, we perform electrochemical impedance spectroscopy (EIS) measurements to examine ionic transport dynamics under varied DC potentials [46, 47]. The EIS data, covering a wide scanning frequency (1000 kHz to 1 mHz), displays a double arc feature and an inductive feature in the fourth quadrant complex plane at higher potentials (Figure S12). The crossover frequency, marking the onset of inductive characteristics, is approximately 20 mHz and increases with applied potentials, corresponding to the frequency range where noticeable hysteresis emerges (Figure 2b). The experimental results align well with an equivalent electric circuit model containing an inductor, confirming the presence of a chemical inductor in our PNCs. The inductive current response (Figures S10 and S13) is consistent with minimal dynamic models [45]. These findings suggest that the chemical inductor, possibly related to the high surface charges of the PNCs, plays a significant role in ionic memory formation, particularly in confined fluidic systems.

### 2.3 Optical Synaptic Plasticity of Nanofluidic Channels

**FIGURE 3:**
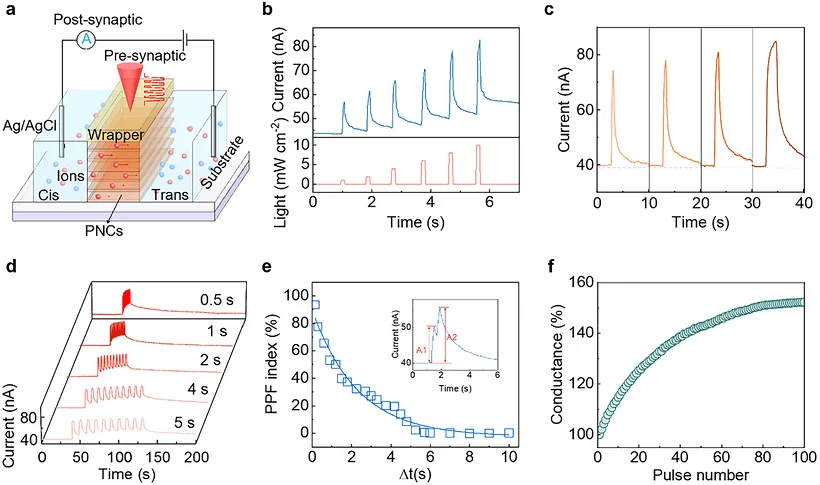
Optical plasticity of the OENFM. (a) Schematic representation of the light‐modulated synaptic plasticity measurements on the OENFMs. The applied optical stimulations operate as the presynaptic signal, and the ionic transport through the PNCs is recorded as the post‐synaptic signal. Altering the optical frequency or amplitude enables the assessment of the optical plasticity of the OENFMs. Post‐synaptic current curves of the OENFMs under (b) different light illumination intensities and (c) varying illumination durations (0.25 s, 0.5s, 1s and 2 s), presented from left to right. (d) Excitatory post‐synaptic current induced by a train of optical pulses with different durations at a fixed light intensity. (e) Optical PPF index as a function of light pulse interval time. The inset illustrates the current output at pair‐pulsed light illumination, with the current ratio (A_2_‐A_1_)/A_1_ denoted as the optical PPF index. (f) Long‐term optical potentiation with 100 pulsed light illumination. All tests were conducted in 1 mM KCl solutions (pH ≈ 6.8).

The photoresponsive characteristics inherent in the photoactive pigments endow the PNCs with the ability to perceive light and respond to programmed light pulses (Figure 3a). Upon exposure to pulsed light illumination (405 nm), an electrical current increase is observed under a minimal bias read potential (50 mV), which subsequently decays and stabilizes to a moderate value after illumination (Figure 3b and Figure S14). The light‐induced current response can be described by a double‐exponential function [43]

$$y\;=y_{0}+y_{1}e^{-\frac{\Delta t}{t_{1}}}+y_{2}e^{-\frac{\Delta t}{t_{2}}}$$

This model, widely applied in optoelectronic systems [23], yields time constants of t_1_ = 0.64 s and t_2_ = 6.99 s, implying rapid and slow decays in the PNCs during light exposure. These distinct decay processes are likely the result of an immediate response from inherent photoconduction [48] and a delayed response caused by surface adsorption [49]. The discrepancy between the final steady current and its initial value is referred to as non‐volatile current [43], a hallmark of optical memory. This long‐lasting non‐volatile behavior is potentially due to the extra proton generated by the photoactive pigment, which contributes to extra charge carriers under light exposure [11, 25].

As the light intensity and duration of illumination increase, both the current variation and nonvolatile current are prominently enhanced (Figure 3b,c), suggesting an alternative approach to modulate the conductance states of the PNCs optically. Intensified light produces more photogenerated carriers, resulting in a higher PSC level. Concurrently, the highly charged surface of extremely confined PNCs impacts ion transport electrostatically, leading to a more pronounced slow decay process. These results suggest that the rapid decay mechanism is primarily associated with photogenerated carriers, likely influenced by illumination intensity, whereas the slow decay mechanism appears to be affected by the duration of illumination. In addition, the PNCs display varying electric currents in response to different light wavelengths, which align well with the corresponding absorption spectrum (Figure S15). It is noteworthy that such optical modulation of ionic memory in fluidic nanochannels is rarely achieved in previous fluidic systems [15, 16, 17, 20, 28, 50].

Light‐regulated ion transport in our system results from photochemical reactions of the light‐responsive molecules within the PNCs. To exclude photothermal effect, we monitored the temperature of the PNCs under illumination (100 mW cm^−^^2^, 405 nm) and observed only a modest ≈2.5 °C increase. Ionic conductivity measured at this elevated temperature revealed no significant change relative to room temperature (Figure S16), ruling out photothermal contributions. Instead, illumination triggers photochemical reactions that release positively charged ions (e.g., Na^+^) into the solution while increasing the negative charge density of resident groups within the PNCs. This synergistic effect enhances the effective surface charge, thereby promoting ion adsorption and transport. Consistently, surface potential measurements under illumination revealed a 17 mV increase (Figure S16), corresponding to a 1.5 mC m^−^^2^ rise in charge density [51], confirming the photochemical origin of the light‐induced ion modulation mechanism.

To explore the short‐term plasticity (STP) under light illumination, the PNCs are exposed to a series of consecutive light pulses at a fixed intensity (50 mW cm^−2^). The resulting PSC consistently improves, leading to a pronounced nonvolatile current (Figure 3d). This facilitation arises from the release and subsequent accumulation of photon‐induced charge carriers in the PNCs, which do not revert to the initial state before the arrival of the next light pulse. As more light pulses are applied, more charges accumulate, implying that the interval between light pulses plays a critical role in current enhancement. Therefore, the frequency of light pulse stimulation is varied to examine this effect. As shown in Figure 3d, at longer optical pulse durations, the PSC almost returns to its initial value, suggesting negligible current facilitation at extended pulse intervals. This is consistent with expectations and further verifies the photon‐induced charge accumulation mechanism. The PPF behavior under light illumination is assessed by applying pairs of light pulses with varying intervals (Figure 3e). As the interval between light pulses decreases, the PPF ratio, defined as (A_2_‐A_1_)/A_1_, notably increases, suggesting a short‐term optical facilitation. These results reveal a gradual decrease in the PPF index, followed by a clear plateau at intervals longer than 5 s. Such dynamics are consistent with those observed in biological optical synapses [43]. The long‐term response is gauged by applying multiple repeated light pulses, where the PSC gradually enhances with more light pulses, suggesting prominent optical LTP (Figure 3f).

### 2.4 Heterosynaptic Plasticity of Nanofluidic Channels

**FIGURE 4:**
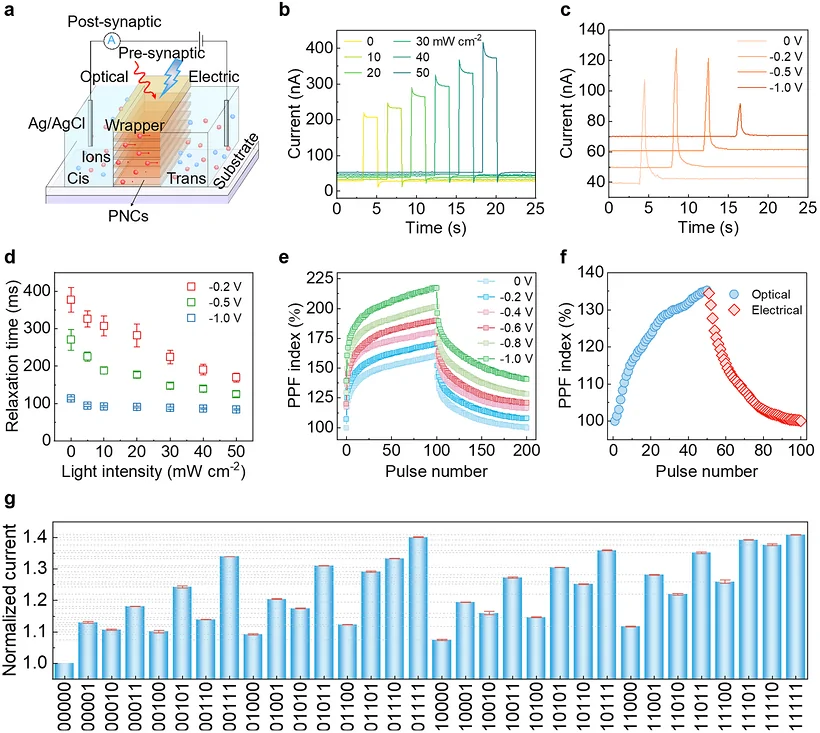
Heterogeneous plasticity of the OENFM. (a) A schematic of the optoelectronic fields modulated heterogeneous plasticity of the OENFMs. The optical or electrical stimuli act as the gate to module the ionic transport through PNCs, with the post‐synaptic current being recorded using redox electrodes. The optoelectronic synaptic response can be evaluated by manipulating the sequence of optical and electrical stimuli. (b) Post‐synaptic current induced by an electrical pulse stimulation (0.4 V, 2 s) under various light intensities (0 to 50 mW cm^−2^). (c) Post‐synaptic current induced by an optical pulse stimulation (10 mW cm^−2^, 1 s) under diverse gate voltages (0 to –1.0 V). The current was measured under a low bias of 0.05 V. (d) Relaxation time extracted from the decay current curves in b, which decreases with increasing light intensity under electrical stimuli. (e) Long‐term electrical potentiation with 100 positive pulses (2 V, 100 ms) and long‐term electrical depression with 100 negative pulses (−2 V, 100 ms) under different gate voltages; a small bias (0.05 V) was utilized to read the conductance state. (f) Optical field‐enabled long‐term potential with 50 sequential pulses (10 mW cm^−2^, 100 ms) for writing information and electrical field‐induced long‐term depression with 50 sequential pulses (−2 V, 100 ms) to erase the encoded information and restore the conductance to the initial state. (g) Current response of the OENFMs under 5‐bit optical pulse trains ranging from (00000) to (11111), leading to thirty‐two fully distinguishable conductance states. The red lines indicate the error bar. All tests were conducted in 1 mM KCl solutions (pH≈6.8).

As previously discussed, we revealed that applying either electrical or optical stimuli can induce diverse synaptic plasticity in the PNCs. This outcome heavily relies on the unique interactions between ion‐charged surfaces in confined space. A more intricate synaptic plasticity is anticipated when these two distinct signals are applied concurrently. The electrical voltage, for instance, can serve as a continuous modulator of the synaptic weight of the PNCs, particularly under simultaneous application of constant light illumination (Figure 4a).

To explore heterosynaptic plasticity, we first examined the electrical response of the PNCs in a three‐terminal transistor under varying light intensities. Notably, gate control in the PNC‐based transistor provides an extra degree of flexibility in modulating the dynamic ionic behavior through the PNCs, thereby promoting electrically adjusted heterosynaptic plasticity. As shown in Figure S17, the PNCs are located between the source and drain electrodes and serve as the central ionic transport channel. The liquid‐gate electrode is positioned adjacent to the PNC region, with a gate‐to‐channel spacing of approximately 200 µm, and the liquid‐gate region is defined by photocured resin boundary. A gate potential was applied to modulate the conductive states of the PNCs through the LiPF_6_ electrolyte rather than directly to the PNCs, thereby minimizing leakage and enabling capacitive modulation of the PNC‐electrolyte interface [52]. Because LiPF_6_ exhibits high resistivity and is insoluble in the aqueous source/drain electrolyte, ion transport within the PNCs remains largely unaffected by electrolyte mixing. As a result, leakage current is negligible under low bias, and the gating response is governed by capacitive charging rather than faradaic processes. Compared with conventional dielectric gates [53], this liquid electrolyte gate provides a simple, low‐leakage, scalable route for fabricating and modulating individual nanofluidic devices, while avoiding the stringent fabrication requirements of ultrathin dielectric layers and reducing the electrochemical complications associated with direct metal contact [13].

As depicted in the transfer curves (Figure S17), the PNCs display a typical p‐type characteristic, consistent with previously reported results [9, 54]. Moreover, the output curves of the PNCs reveal significant modulation of the drain current at negative potentials. The on‐state current at V_g_ = 0 V arises from ion migration driven by the intrinsic surface charge of the PNCs, enabling spontaneous transport through the channel. Such baseline currents, commonly observed in ionic or electrolyte‐gated transistors [52], do not compromise the gating efficacy, as the applied voltage still effectively modulates the channel conductance. We further evaluated the performance of the ionic transistor under light illumination, observing an increase in the drain current at higher intensities (Figure S18). The performance of the ionic transistor is primarily influenced by the interfacial charge state of the PNCs and is therefore strongly affected by electrolyte concentration within the nanochannel [54]. As expected, the baseline ionic current increases with ion concentration. However, the gate‐induced modulation becomes weaker at higher concentrations because stronger electrical double‐layer screening reduces the efficacy of the gate voltage to modulate the surface charge, rendering the ionic current less sensitive to gating. Conversely, at lower concentrations, reduced screening allows the gate voltage to more effectively regulate the interfacial charge density, yielding superior conductance modulation and higher on/off ratios. Importantly, the gating response cannot be described solely by a simple change in surface charge density. In the PNCs, a more negative interfacial potential not only changes the effective surface charge but also strengthens Coulombic attraction between the channel wall and counterions. This can increase local ion occupancy, enhance interfacial ion adsorption, prolong ion residence time, and modify local charge screening within the confined nanochannels. These coupled processes directly affect conductance relaxation and hysteresis. Therefore, the gate‐modulated conductance and memory behavior are better understood as the result of interfacial electrostatic regulation, including surface charge modulation, ion adsorption/desorption, residence‐time variation, and screening effects. Above 1 mM, the decline in ion selectivity within the PNCs aligns with conductivity‐concentration behavior (Figure S8), which displays nonlinear conduction below 1 mM and near‐ohmic characteristics above. Under these high‐concentration conditions, weakened gate control results in a persistent “on” state with minimal regulatory impact. The observed on/off ratio of approximately 5 thus reflects the concentration‐dependent screening effect, with more pronounced gating at lower electrolyte concentrations.

While steric hindrance and dehydration barriers may contribute to ion transport in the PNCs, surface charge effects dominate, as supported by our experimental data (Figure S5) and previous studies [55, 56]. These findings further reinforce the understanding of concentration‐dependent behavior in sub‐nanometer channels. We noticed a concentration‐dependent ratio in the PNCs (Figure S18), with a higher rectifying ratio (≈6) achieved at lower concentrations due to the strong ion‐charged surface interactions from the extended electrical double layer, comparable to previous ion‐based transistors [54, 57, 58, 59]. Although this rectification is not as remarkable as that of solid‐state electronic transistors operated at ultrahigh gate voltages (> 60 V), the extensive tunability through external stimuli and surrounding ions offers a potent alternative approach for adaptation and response to various conditions, which is a challenge with conventional electron‐based transistors. As illustrated in Figure 4b, with consistent electric pulse stimulations, the responsive current of the PNCs notably rises with enhanced light intensities from 0 to 50 mW cm^−2^, and the slight increase in the baseline current is attributed to the individual contribution from light illumination.

To assess the gating effects of the ionic transistor, we applied a range of negative gate voltages, ranging from 0 V to −1.0 V. The experimental configuration in Figure 4a was designed to evaluate the device's multimodal response under simultaneous optical and electrical stimulation, following established methodologies for optoelectronic devices [23]. As demonstrated in Figure 4c, the baseline current increases with gate potentials, paralleling the trend seen in light stimulation under intensified illumination. However, the response current diminishes gradually at higher electric fields. We extracted the relaxation time of the PNCs, defined as the duration required to decay to its 1/e level of the maximum response current at varying gate potentials and light intensities (Figure S19, fitting). Strongly negative gate potentials (e.g., −1.0 V) substantially increase the surface charge density in the PNCs, strengthening electrostatic attraction and trapping of photogenerated carriers. This enhanced interfacial charge also increases local screening and restricts their effective release and transport, thereby suppressing the photocurrent contribution. The suppressed photoresponse at large negative gate biases thus reflects the nonlinear interplay between gate‐induced surface charges and light‐induced carrier transport. Quantitative analysis manifests a shorter relaxation time at higher light intensities or more negative gate bias (Figure 4d). Higher light intensities generate photocarriers (i.e., protons) more rapidly, leading to faster activation and decay. Consequently, a shorter relaxation time is noted at higher illumination intensity and more negative gate bias. The overall synaptic plasticity of the PNCs is determined by the combined effects of both optical and electric signals, making it suitable for multimodal applications. To evaluate long‐term heterosynaptic plasticity under simultaneous optical and electrical gating, we applied trains of electrical pulses at different gate potentials. The PNCs display pronounced LTP and LTD behaviors (Figure 4e), with higher conductivity achieved under stronger gate potentials, suggesting an alternative route for modulating synaptic plasticity. Notably, the currents generated by multiple light pulses can be completely erased by consecutive negative potential pulses (Figure 4f), rendering this approach promising for multimodal information encryption.

The abundant synaptic plasticity modulated by light, electric bias, or their combinations suggests promising applications for the PNCs in neuromorphic computations. We utilized diverse optical pulse trains (5‐bit) to simulate a situation for light pattern recognition. The current response is recorded after a specific optical signal illumination, consisting of five pulses, either on (‘1’) or off (‘0’), with the same pulse duty (2 s, 50 mW cm^−2^) and pulse interval (2 s). Receiving pulse ‘1’ generates an increased current, while receiving pulse ‘0’ yields an unchanged current. Due to the remarkable optoelectronic properties of the PNCs, the output current continuously increases, rather than saturating, over consecutive light pulses (5‐bit) and slowly decays following the cessation of illumination. This can be attributed to the slow dynamics of photogenerated protons and hysteretic ion transport, suggesting that current facilitation/depression and dynamic conductive states with well‐separated outputs can be achieved in the PNCs. As illustrated in Figure 4g, 5‐bit optical pulse trains ranging from ‘00000’ to ‘11111’ produce 32 distinguishable conductance states. Each conductance state was measured five consecutive times across at least three independent devices. The standard deviation of the conductance values remained below 10% for all states, confirming that the optical conductance states are stable, well defined, and reproducible. This indicates a robust capability of mapping complex spatiotemporal signals into separate states, which is essential and beneficial for conducting neuromorphic computations. It should be noted that, in principle, longer bit trains enable encoding of more of preceding states and thus stronger temporal information–processing capability. In short‐memory systems, however, the effective memory length is constrained by intrinsic forgetting or decay. Accordingly, we use a 5‐bit optical pulse train in our simulations, which preserves sufficient temporal information while ensuring that the resulting states remain well separated.

**FIGURE 5:**
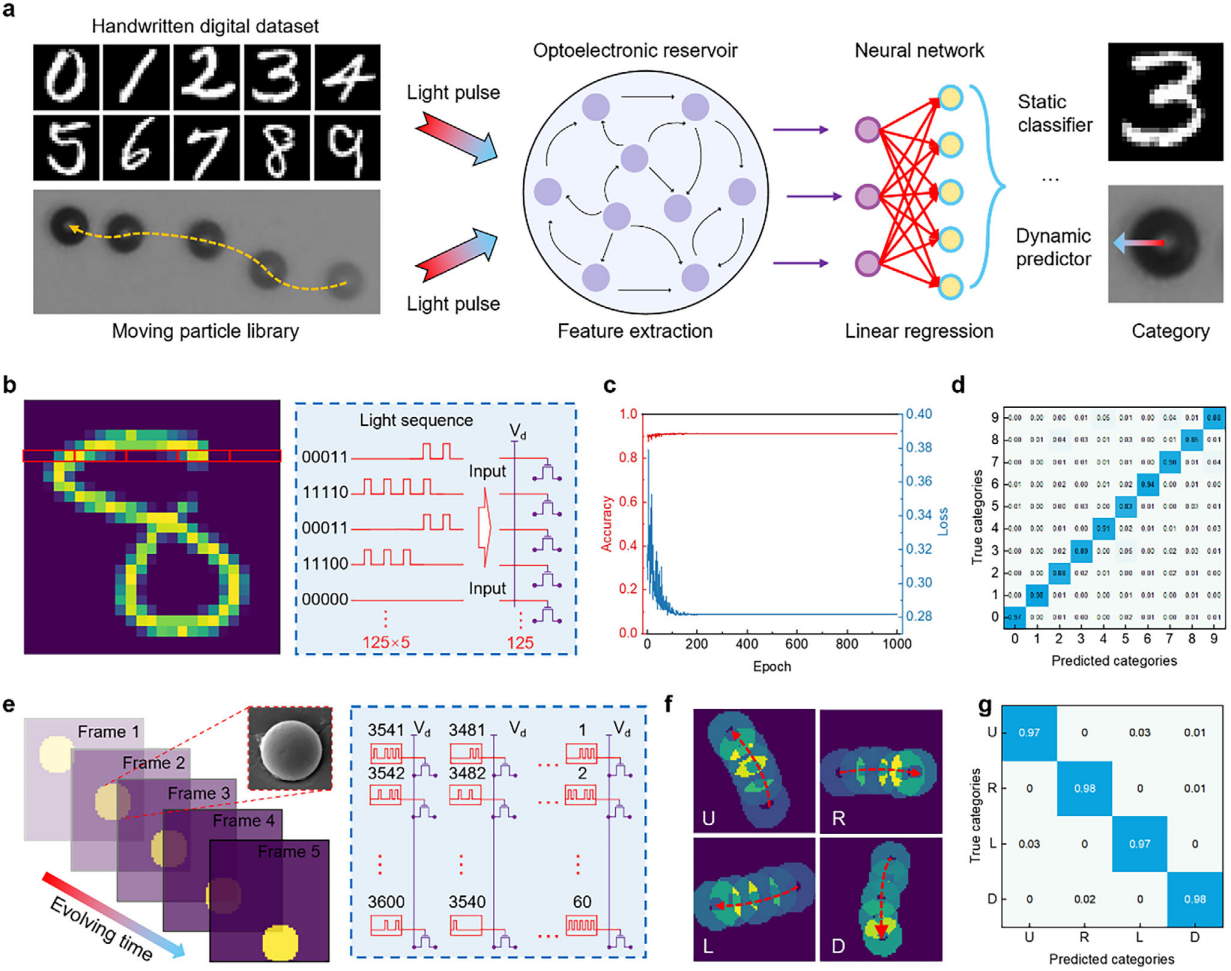
Nanofluidic memristor‐based ionic neural networks for reservoir computing. (a) Illustration of the reservoir computing (RC) architecture based on the optoelectronic nanofluidic memristor (OENFM). The system processes two distinct tasks: static handwritten digit classification and dynamic trajectory prediction of moving microparticles. The OENFM reservoir utilizes its intrinsic ionic memory for spatiotemporal feature extraction, followed by a neural network readout layer to output the final classification or prediction categories. (b) Data preprocessing and encoding strategy for the MNIST dataset classification. The input images are pixelated and mapped into temporal optical pulse sequences using a 5‐bit binary encoding scheme. These sequences are fed into the reservoir via a 125 × 5 input array. (c) The evolution of classification accuracy and loss function over training epochs, demonstrating rapid convergence. (d) Confusion matrix for the recognition of handwritten digits (0–9), indicating high classification fidelity. (e) Schematic of the dynamic trajectory prediction task. A moving microparticle is recorded across five sequential frames mapped to a 60 × 60 pixel grid. The system exploits the device's memory effect to detect the overlap between consecutive frames, enabling the extraction of motion features (e.g., direction) from the evolving temporal input. Inset shows an SEM image of the microparticle. (f) Representative reconstructed trajectory heatmaps corresponding to four movement directions: Up (U), Right (R), Left (L), and Down (D). (g) Confusion matrix for the dynamic trajectory prediction, achieving a recognition accuracy of approximately 97%.

### 2.5 Reservoir Computing Based on Nanofluidic Channels

The non‐linear dynamic current evolution of the OENFM under different optical stimuli (Figure 4g), characteristic of short‐term memory behavior, renders it an appealing opportunity for implementing brain‐like recurrent neural network algorithms, such as reservoir computing (RC) [60, 61]. Unlike conventional neural networks, RC employs a fixed reservoir with random connections to map temporal input data to an output layer, requiring training only for the weights in the readout layer. This results in a significant cost reduction compared to fully connected neural networks, which necessitate extensive training for a substantial number of weights across multiple hidden layers. The completely separated conductance states produced by various light pulse trains in the OENFM are promising for in‐sensor RC implementation (Figure 4g). Figure 5a illustrates the schematic of our optoelectronic RC system, used to perform multi‐task simulations ranging from static image classification to dynamic motion prediction. The recurrent hidden layer, also well‐known as the ‘reservoir’, is implemented directly on an OENFM without additional training. Only the readout weights connecting the reservoir to the output layer are iteratively optimized to minimize system loss. Unlike conventional physical reservoirs, this optoelectronic RC system maps raw temporal optical signals directly into the feature space without digitalization or analogue‐to‐digital conversion, offering a more efficient and cost‐effective route toward edge learning [61].

The capability of static pattern classification of our optoelectronic reservoir is first demonstrated by simulating a standard benchmark based on the Modified National Institute of Standards and Technology (MNIST). The images of 25 × 25 pixels (with blank edge pixels trimmed) are binarized and reshaped into 125 × 5 arrays (Figure 5b). Each set of five spatially localized pixels is then converted into 5‐bit binary light pulses and fed into the OENFM (Figure 5c). Through this encoding process, localized spatial information is transformed into a temporal signal and processed by the optoelectronic RC according to the current evolution rule under the applied light‐pulse patterns (Figure 4g). The state of the reservoir is then transformed into 125‐long visual features and sent to the readout map through neural networks for regression analysis and recognition. As shown in Figure 5c, the recognition accuracy of the MNIST dataset is approximately 91% for distinguishing different digit numbers. We further utilized other public datasets to evaluate the properties of the optoelectronic reservoir, which reveals an accuracy of 97% and 91% for recognizing the letters and the garments from E‐MNIST letters and Fashion‐MNIST datasets, respectively (Figure 5d, Figures S20 and S21). These accuracies exceed those of previously reported fluidic memristors (Table S1), including tapered microfluidic channels [18] (81%) and droplet interface synapses [62] (86.2%). The confusion matrix rows are predominantly dominated by their diagonal elements, suggesting the high class‐wise accuracy of our optoelectronic reservoir on different tasks (Figure S22).

Beyond static image recognition, the unique spatiotemporal dynamics of our optoelectronic reservoir system are particularly advantageous for dynamic motion perception [24]. To demonstrate this capability, a two‐dimensional array (60×60) of our OENFMs is configured to predict the direction of moving particles (Figure 5f). The particles are home‐built photoactive TiO_2_ microspheres (≈5 µm diameter) dispersed in ferrocene solution that functions as a chemical fuel, and are placed in an enclosed transparent chamber for motion investigation. Under UV illumination from different orientations, the particles exhibit distinct trajectory patterns [63]. A commercial camera records their moving trajectories at 25 fps, and the resulting videos are categorized into four motion directions (Up, Down, Left, Right). Each video is clipped into five‐frame sequences and augmented by rotations of 90°, 180° and 270°, producing a dataset of 1920 samples. After binarization (0 for dark, 1 for light), these frames (with 60 × 60 pixels) are fed into the optoelectronic reservoir to extract spatiotemporal features (Figure 5f).

As shown in Figure 5g, after physically processed by the OENFMs, the extracted feature exhibits a noticeable overlap between past and present frames, reflecting the device's short‐term memory and the distinct spatiotemporal signatures associated with each motion direction. These features are subsequently fed into a linear classifier (implemented on a computer) to determine the particle's direction. The accumulative photochemical response of the device imprints memory of preceding frames onto the current state, enabling reliable prediction of future motion. The trained neural network achieves a 97% testing accuracy in determining the actual movement direction, with the confusion matrix showing uniformly high class‐wise performance (Figure 5g and Figure S23). It should be noted that, when implementing reservoir computing with OENFMs, the chemical environment, including electrolyte composition, pH, and temperature, is typically fixed to ensure stable and reproducible ionic relaxation dynamics [61]. Under these controlled conditions, OENFMs provide reliable fading memory and nonlinear responses for physical reservoir computing [60]. Meanwhile, this environmental sensitivity also offers a functional advantage for nanofluidic systems, as tunable physicochemical relaxation processes can serve as additional degrees of freedom for regulating reservoir dynamics and enabling adaptive ionic computing. This feature distinguishes nanofluidic ionic reservoirs from conventional solid‐state electronic reservoirs, whose behavior is mainly governed by electron or hole transport [60]. In complex biomedical applications, such as targeted drug delivery [64, 65, 66], precise motion prediction is critical, and the ultrahigh recognition accuracy of our optoelectronic reservoir offers an efficient pathway for real‐time movement analysis and trajectory control.

### 2.6 In‐Sensor Computing of Nanofluidic Channel Arrays

**FIGURE 6:**
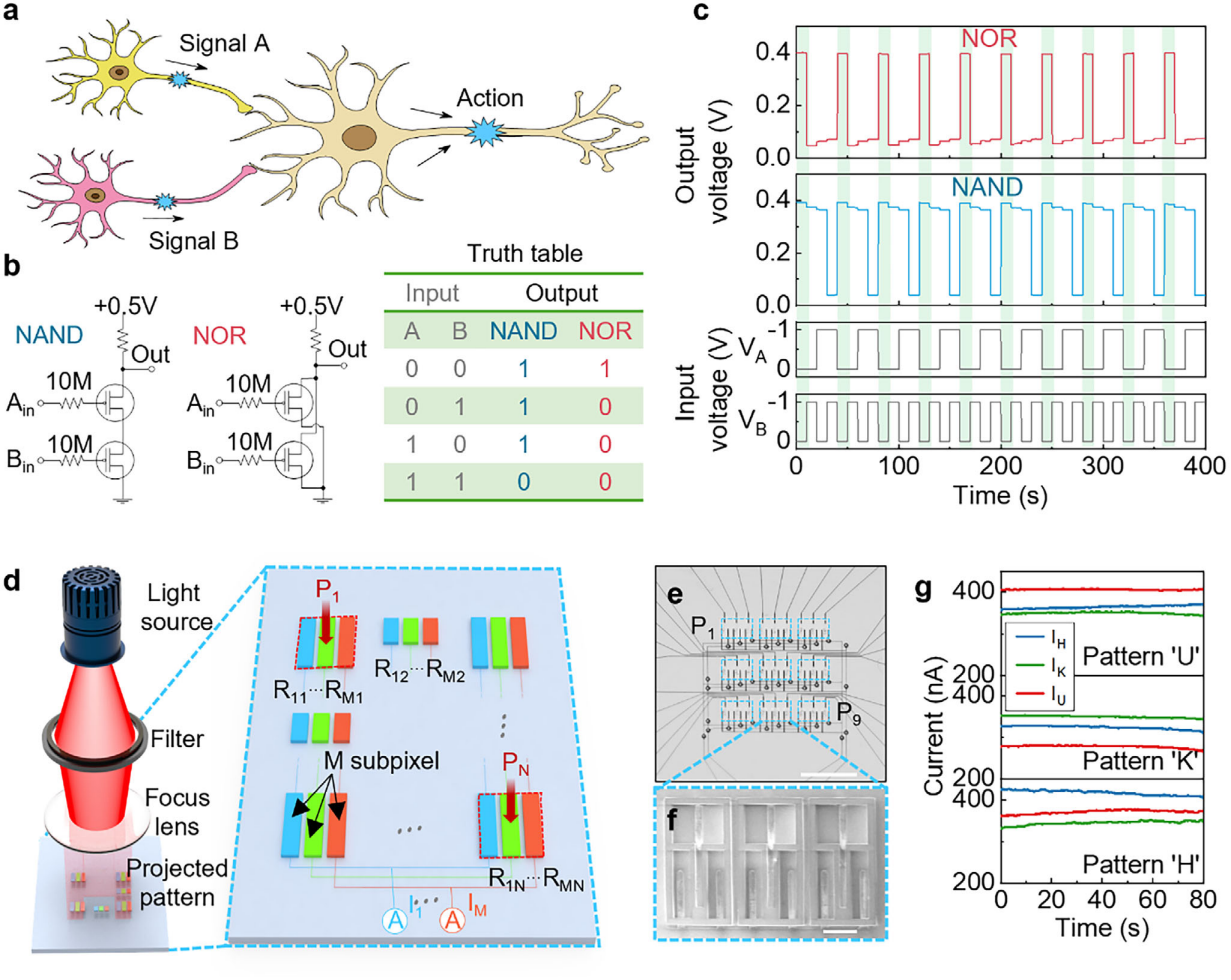
Bio‐inspired logic operations and in‐sensor computing implementations using the nanofluidc memristor‐based ionic neural networks. (a) Schematic illustration of biological signal integration in a neural network, where dendritic signals (Signal A and B) effectively summate to trigger a somatic action potential, serving as the biological inspiration for ionic logic. (b) Circuit configurations for constructing ionic logic gates. A NAND gate is realized by connecting two OENFMs in series, while a NOR gate is formed by connecting them in parallel. The corresponding truth table validates the logic states (0 and 1) for inputs A and B. (c) Experimental dynamic output voltage characteristics of the ionic NAND and NOR gates. The curves demonstrate robust logical switching in response to square‐wave input voltage sequences (V_A_ and V_B_), consistent with the truth table. (d) Schematic of the optoelectronic in‐sensor computing system. A rewritable projector casts light patterns onto the OENFM array. The array consists of multiple pixels (P_1_ to P_N_, where N = 9), where each pixel comprises M addressable subpixels (M = 3) acting as tunable weights. The currents from specific subpixels are summed according to Kirchhoff's law (I_1_…I_M_) to perform vector‐matrix multiplication directly within the sensor. (e and f) Optical and scanning electron microscopy images of the ionic neural networks (3 × 3 pixels) and one enlarged pixel (3 subpixels). Each pixel (P_1_, … P_9_) consists of three OENFM subpixels with responsivities set by the gate voltages. Scale bars, 5 mm (e); 500 µm (f). (g) Experimental demonstration of optical pattern recognition for the letters ‘H’, ‘K’, and ‘U’. The system outputs three distinct current channels (I_H_, I_K_, I_U_). The results show that the output current corresponding to the projected target letter (e.g., I_U_ (red line) for Pattern ‘U’) consistently exhibits the highest amplitude, confirming successful in‐sensor classification.

The multimodal responsiveness of the OENFMs facilitates not only effective deployment as a physical reservoir for executing versatile RC but also enables the integration of computational capabilities directly into the intrinsic optoelectronic sensors, allowing for real‐time data processing and on‐site computation. Inspired by the human brain, the information received by the adjacent neuron relies on input signals from surrounding neurons, which perform Boolean logic computations to initiate specific actions (Figure 6a). Therefore, the successful scaling and integration of multiple memristors are a vital prerequisite for executing the above‐mentioned computations; however, they are considerably challenging in aqueous ionic systems due to the amorphous fluidic features. The PNCs demonstrated here, characterized by their planar configuration, compact size, and simple structure, enable easy and effective scalable fabrication and integration, eliminating the need for complex channel engineering, intricate fluidic networks, and bulky reservoirs. To demonstrate the concept's validity, we first fabricated and connected two OENFMs to establish ionic logic circuits, which are the basic building blocks for conducting complex computations in iontronics. As illustrated in Figure 6b, two gate‐controlled OENFMs connected with appropriate resistors generate the “NAND” and “NOR” ionic logic gates. The truth table for these ionic logic gates is presented in Figure 6b, where a binary “1” signifies a high value and “0” is a low status, applicable to both input and output signals. Two voltage inputs (A_in_ and B_in_) are fed to the ionic logic circuit, and the resulting outputs (V_out_) are recorded. In the “NAND” gate, a low output voltage of approximately 0.04 V is attained when two high signals (−1 V) are input, while other scenarios yield a high output signal (≈0.4 V) with minor fluctuations (Figure 6c). In contrast, the “NOR” gate exhibits a high output voltage of about 0.4 V when both inputs are low (0 V), with V_out_ decreasing to a lower value (0.04–0.07 V) in other cases. These results confirm the successful construction of ionic logic gates, highlighting their functionality and potential for constructing ionic integrated circuits.

In contrast to conventional electron‐based memristive devices, the OENFM showcases a tunable photoresponsivity, which can be modulated by adjusting the gate voltages (Figure S24). By applying a specific bias to the gate voltage, we were able to adjust the photoresponsivities from 4.1 to 10.5 mA W^−1^. This tunable photoresponsivity sets the OENFM apart from previous semiconductor‐based devices, which typically display a constant responsivity determined by a fixed bandgap. Moreover, the optical memory of the OENFM is dependent on the pulse duration; a shorter pulse leads to a transient memory, making it ideal for implementing in‐sensor computing(Figure S25) [67, 68, 69]. An OENFM array can be well designed to facilitate real‐time in‐sensor convolution of a projected image with a photoresponsivity matrix, where each element's matrix value can be individually optimized through ANN training (Figure 6d, and Figure S26).

To illustrate this concept, we further fabricated an OENFM array consisting of 27 subpixels, with the interconnection layout shown in Figure S26. The array displayed considerable uniformity and performance tunability, with both the electric output and optical response being highly concentrated and narrowly distributed (Figure 6e, f, and Figure S27). The rectification ratio extracted for each device in the array exhibits a narrow distribution, with a coefficient of variation below 15% (Figure S28), demonstrating excellent array‐level uniformity. In principle, larger arrays with higher integration numbers and device densities can be fabricated using this approach, as the nanofluidic memristors are prepared by scalable solution processing combined with standard microfabrication techniques. The photocurrent (I^p^) from individual units under optical illumination, which is defined by subtracting the dark current from the total current, was summed according to Kirchhoff's law when arranged in parallel. Each subpixel was connected to an individually addressable gate, enabling tunable photoresponsivity. This configuration allows the device to distinguish different letters (H, K, U) encoded as 3 × 3 pixel patterns. Optimized weights, i.e. the desired photoresponsivity values, were obtained through ANN training on a computer (See details in Methods). These values were then mapped to gate voltages according to the relationship in Figure S24 and applied to the OENFM array via a home‐built peripheral circuit (Figure S26). Letter patterns were projected onto the device using a commercial digital light projector, and the resulting output currents reliably identified the projected letters. The well‐separated current levels (Figure 6g), consistent with the simulated outputs (Figure S29), confirm robust inference capability. Although expanding the network size and shrinking subpixel dimensions could potentially enhance performance, these remain notable fabrication challenges. In terms of energy efficiency, each computing event consumes 5.5–27.5 nJ, comparable to state‐of‐the‐art fluidic neuromorphic devices [24, 70, 71]. Miniaturization and channel engineering should further reduce consumption. We anticipate that the nanofluidic memristor could be integrated into a self‐powered sensation‐computation all‐in‐one system by incorporating gradient electrolytes as the power source [11], akin to a biological retina, which could potentially pave the way for more cost‐effective and efficient neuromorphic computing.

## 3 Conclusions

Ionic machinery that mimics biological systems through inherent sensation and adaptive memory is highly desirable and offers significant potential for neuromorphic computing. In this study, we have engineered a novel ion‐based OENFM that exhibits multimodal stimuli perception and hysteretic conductance in aqueous solutions. The OENFM rapidly responded to a broad spectrum of electrical, optical, and optoelectronic signals. The resulting ionic memory can be conveniently modulated by modifying the amplitudes, frequencies, durations, and intervals of the imposed signals. Experimental investigations and spectral measurements revealed that the surface ion adsorption and chemical inductors, related to the high surface charges, contribute to the formation and evolution of the non‐trivial ionic memory within OENFMs. Strikingly, by changing electrolyte concentrations, ionic types, or pH levels in the aqueous solution, we achieved varied chemical modulation of the OENFMs’ synaptic memory, a feat challenging to achieve in conventional electron‐based solid systems. Therefore, our OENFMs demonstrated a range of synaptic plasticity, including STP, STD, LTP, LTD, PPF, and PPD. This robust synaptic plasticity enabled the successful operation of RC to recognize different image patterns from the MNIST datasets and perform dynamic motion recognition from a homemade moving particle library with a high accuracy of about 91% and 97%, respectively. Additionally, the OENFMs have been produced on a large scale and effectively integrated into an image sensor that responds to external light and performs pattern classification simultaneously, with satisfactory differentiation.

To the best of our knowledge, this work presents the first multifunctional ionic neural network operating entirely in aqueous environments. Table S2 benchmarks our device against representative fluidic devices, underscoring the unique combination of multimodal synaptic modulation and system‐level neuromorphic integration achieved by our OENFM. Although the current image sensation rate is slower than biological vision, increased device density and further miniaturization of feature dimensions are expected to substantially improve throughput. The present operation under blue illumination is primarily determined by the photoactive molecules used; integrating photoresponsive materials with broader absorption spectra and higher quantum efficiencies offers a clear route toward full‐spectrum functionality. The response speed is currently constrained by intrinsic ion transport dynamics in confined channels. Performance could be enhanced through reduced channel length, optimized interlayer spacing, electrolytes with higher ion mobility, and strategically engineering operating environments. Overall, the demonstrated ion‐mediated nanofluidic architecture establishes a promising platform for neuromorphic electronics. We envision integration with power‐supplying, sensing, and actuation modules to realize fully autonomous ionic computing systems capable of complex, real‐world perception and computation.

## Conflicts of Interest

The authors declare no conflicts of interest.

## Supporting information




**Supporting File**: adma74080‐sup‐0001‐SuppMat.docx.

## Data Availability

The data that support the findings of this study are available from the corresponding authors upon reasonable request.
