# Supplementary material for: Optoelectronic Nanofluidic Neural Networks for Ionic Computing
Source: Adv Mater. 2026 Jul 16;38(47):e74080. doi: 10.1002/adma.74080 (PMC13496060; doi:10.1002/adma.74080)
Supplement: Supplementary file 1 [file ADMA-38-e74080-s001.docx]

Supporting Information

Optoelectronic Nanofluidic Neural Networks for Ionic Computing

*Yaxin Huang^1,2#^, Zhiyuan Du^3#^, Changjin Wu^2^, Binglin Zeng^2^, Yingnan Cao^2^, Mingliang Li^2^, Liren Zhang^2^, Qingxin Guo^2^, Chao Huang^2^, Can Li^3*^, Jinyao Tang^2,4,5,6*^*

^1^ School of Mechanical Engineering, Beijing Institute of Technology, Beijing, 100081, P. R. China

^2^ Department of Chemistry, The University of Hong Kong, Hong Kong, 999077 P. R. China

^3^ Department of Electrical and Electronic Engineering, The University of Hong Kong, Hong Kong, 999077 P. R. China

^4^ State Key Laboratory of Synthetic Chemistry, The University of Hong Kong, Hong Kong 999077, China

^5^ HKU-CAS Joint Laboratory on New Materials and Department of Chemistry, Hong Kong 999077, China

^6^ Materials Innovation Institute for Life Sciences and Energy (MILES), The University of Hong

Kong, Hong Kong, 999077 China

Corresponding Email: canl@hku.hk and jinyao@hku.hk

^#^ These authors contributed equally to this work

**Table of Contents**

[1.	Experimental methods	2](#_Toc228604881)

[Fabrication of photoresponsive nanochannels (PNCs)	2](#_Toc228604882)

[Electric measurements	2](#_Toc228604883)

[Fabrication and measurements of photoresponsive array	3](#_Toc228604884)

[Raman spectroelectrochemistry	3](#_Toc228604885)

[Moving particle library	4](#_Toc228604886)

[Reservoir simulation in extracting features of 2D Images	4](#_Toc228604887)

[Reservoir simulation for 3D particle motion direction recognition	5](#_Toc228604888)

[In-sensor computing for image classification	5](#_Toc228604889)

[2.	Theoretical analysis of electrical response	6](#_Toc228604890)

[Analysis of ionic hysteresis in the PNCs	6](#_Toc228604891)

[Analysis of chemical inductive current	7](#_Toc228604892)

[3.	Supplementary figures and tables	8](#_Toc228604893)

[Reference	39](#_Toc228604894)

# 1. Experimental methods

## Fabrication of photoresponsive nanochannels (PNCs)

Graphene oxide was synthesized from natural graphite powder（325 mesh）using a modified Hummer's method as previously reported^[1]^. The graphene oxide solution (GO, 1 mg mL^-1^) was then mixed thoroughly with photoacid solutions (pyranine, Sigma) to prepare photoresponsive nanochannels (PNCs). Silicon substrate (300 nm oxidized layer) was carefully cleaned by sonication in acetone, DI water, and isopropanol for 5 min, respectively, and dried with N_2_. Subsequently, a standard RCA clean (5:1:1 H_2_O: H_2_O_2_: NH_4_OH, 30 mins) was used to remove the organic contaminants. The silicon substrate was washed in DI water and thoroughly dried with N_2_ for further use. Photoacid-doped graphene oxide (PAGO) solution was cast onto the treated silicon substrate with predefined width (2 mm), and then dried at 95 ℃. Excess photoacid molecules were removed by washing the film three times with ethanol and DI water. To improve the film's stability, a low-temperature annealing process was carried out at 95℃ for 1 hour. Then, maskless photolithography (Smart Print UV, Microlight3D) was conducted on the film to construct nanochannels. The formed PAGO film was first treated in a HDMS chamber for 1 min at 95 ℃ to promote the interfacial contact with photoresist. Negative photoresist (SU-8 3050, Kayaku) was spin-coated to the film and cross-linked by UV light exposure. After development, excess PAGO film not covered by the photoresist was removed by oxygen plasma etching. Meanwhile, the cross-linked photoresist formed two miniaturized reservoirs for electrolyte solutions. The dimension of the PNCs could be easily manipulated by modifying the exposure patterns and adjusting the film thickness, respectively. For a typical measurement, the typical PNC was in a dimension of about 200 µm × 50 nm × 2 mm in length, height, and width, respectively.

## Electric measurements

Electrical measurements (I-V, I-T, I_d_-V_g_, and I_d_-V_d_) of the PNCs were carried out using a high-precision source meter (Keithley 2635B and 2400). The fabricated planar OENFM was filled with electrolytes of varying concentrations (0.001 mM to 1 M) , pH values (acidic to base), and ionic types (K^+^, Na^+^, Li^+^). Homemade Ag/AgCl electrodes were used to apply various potentials, including periodical, biased, sequential, and pulsed signals, across the PNCs and recorded the resulting ionic current. For periodical I-V measurements, sine or triangle potentials of various frequencies (1 to 1000 mHz) were applied, accompanying a sampling rate of 5 Hz. For pulse or sequential measurements, the applied potentials were generated by MATLAB, with specific pulse durations and intervals, and the sampling frequency was set to 50 Hz. Unless otherwise specified, the main I-V and hysteresis measurements were performed at a fixed scan frequency of 10 mHz. LED bulbs (405 nm) were used for light-driven measurements, and the power density was measured by a power meter (Sper Scientific 840011). The illumination time and intensity were controlled by a function generator (FY6900), which facilitated pulse or periodic light stimulation. Temperate-varying measurements were performed using a commercial heater placed beneath the PNCs, with the real-time temperature recorded by a high-sensitivity thermometer. To evaluate the efficacy of the OENFM, a control device without PNCs was constructed. In contrast, the measured current in the PNCs was several orders of magnitude lower than the blank counterpart, confirming the robustness of our OENFM and ruling out leakage through the channel walls.

## Fabrication and measurements of photoresponsive array

A custom-made flexible double-layered PCB (polyimide, 80 µm in thickness) with predetermined gate, source, and drain electrodes as well as electric circuits, was fabricated using standard PCB fabrication procedures. This PCB was thoroughly rinsed with acetone, isopropanol, and DI water three times. It was further treated with oxygen plasma for 1 min to enhance the surface hydrophilicity. The PNC array was then constructed by spraying a PAGO and ethanol mixed solution (3:1 volume ratio) onto the PCB using a shadow mask at 150 ℃. Then, a photolithography process, the same as above, was performed to define the reservoirs.

To measure the performance of the PNC array, a commercial 3LCD projector, and Arduino controllers were utilized to project pre-designed patterns onto the array and to apply an adjustable gate voltage to fine-tune the photoresponsivity, respectively. To narrow the output wavelength range, a blue optical filter was positioned between the dichroic combiner cube and the light source, and the lens was inverted to project high-resolution images. The gate voltage of each sub-pixel can be precisely altered through the PWM method in LabVIEW software. The output current from the array was recorded using a high-precision source meter (Keithley 2635B).

## Raman spectroelectrochemistry

In-situ Raman spectroscopy was carried out on the PNCs using a Horiba LabRAM HR Raman spectrometer (Witec 300 Alpha R) equipped with a 532 nm laser. The laser was focused onto the PNCs using a 50× objective lens, and the laser power at the sample behind the objective was set to 1 mW to avoid sample damage. Electrochemical measurements were performed using a potentiostat (MultipalmSens4), connected to an electrochemical cell filled with a 1 mM KCl electrolyte solution. The potential was scanned over a range of values, with each potential level maintained for 30 s, and the resulting current was recorded.

## Moving particle library

The synthesis of the monodisperse light-driven TiO_2_ microsphere was according to a modified sol-gel procedure^[2]^. Typically, 0.56 g dodecylamine was dissolved in a mixture solution containing 0.24 ml DI water, 100 ml acetonitrile, and 200 ml methanol. After vigorous stirring, 2 ml titanium (IV) isopropoxide was added to the solution, which was then stirred continuously overnight to obtain a homogeneous suspension. The suspension was centrifuged, rinsed with ethanol, and dispersed in 30 ml ethanol to obtain monodisperse TiO_2_ microsphere. To prepare the moving particle datasets, the TiO_2_ was dispersed in ferrocene solution and placed in an enclosed chamber. Upon UV light illumination, the microspheres were propelled, resulting from photochemical reactions occurring at their imposed surface^[3]^. The direction of movement was controlled by altering the light directions. A commercial camera, set at a frame rate of 25 fps, was used to record the trajectory of the microspheres. The moving particles were categorized into four directions with a total number of 480, where the frame size is 60 × 60. After rotated by 90°, 180° and 270°, a dataset with totally 1920 samples were built.

## Reservoir simulation in extracting features of 2D Images

The images from pattern datasets were first binarized into a 5-bit optical sequence, where each timestep was either ‘1’ indicating 2 seconds of light, or ‘0’ denoting 2 seconds of darkness. The PNCs were illuminated ten times, and the respective current was recorded for each type of sequence. Subsequently, a 5-bit optical pulse was fed into the nanofluidic reservoir as the input, and one of the ten responses was randomly selected as the output. The feature extraction results in a 125-long sequence, which was then fed into a neural network-connected readout layer. The weights of the readout map were trained using backpropagation, guided by the experimentally obtained potentiation and depression characteristics. The categorical cross-entropy loss was minimized by the batch gradient descent (batch size = 20) using the Adams (learning rate = 0.01) optimizer. The readout layer was trained 1000 epochs on each independent task. The datasets were divided into training and test sets, with an 6-1 split for training model performance evaluation, respectively.

## Reservoir simulation for 3D particle motion direction recognition

A hundred and twenty shots were captured for each class of moving particles, resulting in a total of 480 records. After rotated by 90°, 180° and 270°, a dataset with totally 1920 samples were built. For each recording, five equidistant frames were selected to form a dataset, and each frame was resized to a uniform 60 × 60 pixels before being binarized. To construct the dataset for the simulated in-sensor RC system, we merged the five frames from each shot in chronological order to create a 3600 × 5 matrix. In this matrix, each row’s pulse sequence corresponded to one of the 32 illumination patterns, ranging from “00000” to “11111”. The in-sensor RC system consisted of 3600 optically operated memory devices that functioned as reservoir nodes. A fully connected readout layer with 3600 inputs, corresponding to the reservoir nodes, and four output neurons representing different motion directions (“up”, “down”, “left”, and “right”) was implemented in the software. The model was trained by minimizing categorical cross-entropy loss using the gradient descent method optimized with the Adam algorithm (with a learning rate set at 0.01). The training process spans 100 epochs. Lastly, the data was partitioned into training and testing sets, with 75% of them reserved for training and the remaining 25% for model performance evaluation.

## In-sensor computing for image classification

The scheme outlined in Figure S26 demonstrates the circuit configuration designed for performing in-sensor computing, which enables the real-time classification of diverse optical patterns. This setup consists of 27 photoresponsive devices, organized into three 3×3 subarrays, with each subarray tasked with differentiating between various optical patterns. Each pixel within the subarray functions as a phototransistor of which the output current can be described as: $I_{mn}=R_{mn}*P_{n}+I_{mn}^{b}$, where *P*_n_ is the local optical power at the *n*_th_ pixel, *R*_mn_ and $I_{mn}^{b}$ are the photoresponsivity and dark current of the subpixel, respectively. With a constant driving voltage, *V*_dd_, both *R*_mn_ and $I_{mn}^{b}$ can be controlled individually by adjusting the gate voltage, Vg. An image array then is formed by interconnecting the subpixels together (Figure S26) and the photocurrent of the *m*_th_ sub-array reads

$$I_{m}^{p}=I_{m}-I_{m}^{b}=\sum_{n=1}^{N} R_{mn}P_{n}, m=1,2,\ldots$$

where $I_{m}^{p}$is the photocurrent calculated by subtracting the dark current $I_{m}^{b}$ from the total output current $I_{m}$. This implements a typical matrix-vector product (MVP) operation *I=RP* in a neural network, with *R*(*R*_mn_) the photoresponsivity matrix, *P*=(*P*_1_, *P*_2_, …, *P*_n_)^T^ the optical image vector projected on the array, and I=(I_1_^P^, I_2_^P^, …, I_m_^P^)^T^ the photocurrent vector. As a demonstration, a neural network was first trained on the above configuration to get the optimal gate voltages, which facilitate to tune the devices’ photoresponsivity for distinguishing between different optical patterns (such as the letters “H”, “K”, and “U”). For the training, a total of 540 binary images (180 for each letter) composed of 3×3 pixels were created in software to construct the dataset with 60% for training and the remaining for testing. Gaussian noise with a standard deviation of σ = 0.01 was added for augmenting the data. A fully connected layer, consisting a 9×3 weight matrix, was established. Softmax functions were utilized as activation functions to project the output to three labels, which were encoded using the one-hot method. The model was trained by minimizing categorical cross-entropy loss using the gradient descent method, with a learning rate set at 0.05. After 100 epochs of training, the optimized weights, i.e. photoresponsivity values were mapped to the gate voltages according to the relationship shown in Figure S24. And then these optimized gate voltages were applied to the fabricated OENFM array through a homebuilt peripheral circuit. Under the letter patterns projected by a commercial projector, the output currents from 3 subarrays were recorded to indicate the inferenced labels. The resultant experimental results are presented in Figure 6g.

# 2. Theoretical analysis of electrical response

## Analysis of ionic hysteresis in the PNCs

The hysteretic conductance in the PNCs is attributed to the intense interfacial process on ions within a confined channel carrying high surface charges. Notably, in our PNCs, the shape of the current-voltage (I-V) and conductance-voltage (G-V) curves under a periodic sinewave potential excitation provides significant insight. The I-V curve displays a self-crossing point, while the corresponding G-V plot depicts an open loop, indicating a bipolar memristor rather than a unipolar memristor of the PNCs. To account for this observed memory behavior in our PNCs, a simplified surface adsorption-desorption model^[4-5]^ is adopted.

The memristor, fundamentally a resistor with a history-dependent conductance, can be represented by the following equations:

$$I=G\left[ n\left( t \right) \right]\Delta V(t)$$

$$\dot{n}=f(n, \Delta V\left( t \right))$$

Here, I denotes the electrical current through the PNCs under a time-varying voltage drop$\Delta V\left( t \right)$, G represents the conductance that is associated with the internal parameter n, which governs the system’s memory and evolves through a voltage-dependent dynamic process, and the dot indicates the time derivative. To interpret the ion transport in the PNCs, a simplified advection-diffusion model is introduced, and the extracted time-varying conductance can be described as:

$$G\left( t \right)= \int_{0}^{+\infty} G_{qs}[\Delta V\left( t-s \right)]\frac{e^{-s/\tau}}{\tau}ds$$

with $G_{qs}$ denotes the quasistatic conductance, where the voltage remains at a specific value $\Delta V\left( t-s \right)$ for an infinite amount of time. It can be expressed as:

$$G_{qs}(v)=G_{0}(1+\beta v)$$

where $G_{0}$is bulk conductance and $\beta$is the parameter defining the adsorption-desorption process. By combining these equations, the experimental I-V results in Figure 1c in the main text can be fitted, and the memory time of the PNCs can be extracted. It should, however, be noted that normalization of the loop area is a prerequisite before the fitting process. For simplicity, the I-V curve was normalized using a rectangle shape formed between the lowest conductance, highest conductance, and the scanning potential, as shown in Figure S9.

## Analysis of chemical inductive current

To comprehend the inductive current observed in our experiments, we employed a simplified model^[6]^ incorporating several equations:

$$V_{a}=R_{s}I_{t}+u$$

$$I_{t}=\frac{u}{R_{H}}+ x\frac{u}{R_{L}}+C_{m}\frac{du}{dt}+C_{P}\frac{dx}{dt}$$

$$\frac{\tau_{k}}{x^{n}}\frac{dx}{dt}=\left( 1-x \right)-e^{-(u-V_{R})/V_{m}}x$$

Here I_t_ is the electrical current and V_a_ is the applied potential. R_s_ signifies the series resistance of the setup while u denotes the voltage across the PNCs. R_H_ and R_L_ are the resistances of the high and low resistance state, respectively. X is a state variable that determines the charging of the PNCs from a low charge state at negative voltages (x=0) to a high conductivity state at positive voltages (x=1). C_m_ represents the channel capacitance, while C_p_ is the capacitance related to the internal wall charging. τ_k_ is the kinetic constant for the establishment of the high conductance state, and the factor τ_k_/x^n^ induces a nonlinear relaxation time dependent on a cooperativity exponent n. V_R_ is the threshold voltage and V_m_ is a steepness parameter. In the steady state, we can obtain

$$I_{tot}=\frac{u}{R_{H}}+ x_{ss}\frac{u}{R_{L}}$$

$$x_{ss}=\frac{u}{1+e^{-(u-V_{R})/V_{m}}}$$

These equations elucidate the observed hysteresis in Figure 1c and the inductive current in Figure S10.

# 3. Supplementary figures and tables

**Figure S1. Fabrication and characterization of the photoresponsive nanochannels (PNCs).** Schematic illustration for the assembly process of (a) the PNCs and (b) the PNC-based ionic device. (c-e) Cross-sectional SEM images of the PNCs with different thicknesses. All the PNCs exhibited uniform interlayer structure, indicating the successful construction of the nanochannels. (f) Top-viewed SEM images of the etched nanochannels covered with cross-linked photoresist. (g) AFM measurements of the PNCs, which reveal a thickness of about 50 nm. (h) Surface morphology characterization of the PNCs, which reveals a roughness of ~3.6 nm.

**Figure S2. Chemical and structural characterizations of the PNCs before and after functionality.** (a-d) XRD patterns, Raman, EDS, and FT-IR spectrum for the pristine nanochannel and modified PNCs.

**Figure S3. Memory time measurement of the PNCs.** (a) Graphical representation of the applied voltages on the PNCs as a function of time. The electrical conductance of the PNCs is measured by imposing a high positive potential (1 V, 40 s) and recorded by a sequence of lower read bias (0.05 V) to avoid potential electrical inference. (b) Relative conductance changes of the PNCs over a specified period of time. The electrical conductance of the PNCs is normalized by dividing the initial conductance before pulsed potential stimulations. (c) Alternating positive and negative voltage pulses were applied to switch the conductance state of the PNCs, with a small read bias of 0.05 V used for conductance readout. (d) Cyclic conductance response of the PNCs under repeated bipolar switching.

**Figure S4. Electrochemical Raman spectrum of the PNCs.** (a) Setup for in-situ spectroelectrochemical Raman measurements. (b) Raman spectrum of the PNCs. (c) Spectrum mapping of the PNCs under a variety of electrical potentials.

**Figure S5. Electrical measurements of pristine graphene oxide nanochannels (NCs).** (a) Current-voltage curves of the PNCs under varying scanning frequencies demonstrate an enhanced hysteresis at lower frequencies. (b) Memory capacity dependence on the scanning frequency. (c) Representative Arrhenius-like behavior of ionic conductance of PNCs at different temperatures.

**Figure S6. Ion adsorption characterization of the PNCs.** (a) Schematic illustration of electrochemical quartz crystal microbalance measurements on the PNCs. The PNCs are immersed in 0.1 M KCl electrolytes, and the frequency response during constant electrical potential is recorded using a highly sensitive quartz crystal. (b) Frequency response of the PNCs under a constant negative voltage over an extended measurement time. (c) Frequency response of the PNCs under varied temperatures. (d) Frequency response of the PNCs under a voltage cycle.

**Figure S7.** **Memory effect of the PNCs at triangle waveform scanning potentials.** (a) Diagram of the scanning waveform on the PNCs. (b) Current-voltage curves of the PNCs under varying scanning frequencies. (c) Memory capacity dependence on the scanning frequency.

**Figure S8.** **Electrical measurements of the PNCs.** (a) Current-voltage curves of the PNCs at different KCl concentrations. Due to the uniform channel structure, the PNCs exhibited slight ion rectifying effect. (b) Calculated ionic conductance of PNCs at different KCl concentrations. The linear dotted line indicates the conductance of the bulk solution. (c) Current-voltage curves of the PNCs at different channel thicknesses. (d) Calculated conductance that shows linear dependence on channel thickness.

**Figure S9.** **Modulation of the PNCs’ memory capacity.** (a) Memory capacity normalization defined by the enclosed area of the I-V curves. (b) Normalized memory capacity of the PNCs under different KCl electrolyte concentrations. (c) Memory capacity of the PNCs with different cation types at 10 mM concentrations. (d) Normalized memory capacity of the PNCs under different electrolyte pH values, adjusted using either KOH or HCl solutions. (e) Zeta potentials of the PNCs at different pH conditions. (f) Normalized memory capacity of nanofluidic memristor under cyclic measurements.

**Figure S10.** **Pulsed electrical response of the PNCs.** I-t curves of the PNCs at single (a) positive and (b) negative pulses. (c) Consecutive electrical response of the PNCs at increased pulse potentials. A noticeable peak in the reversal responsive current is observed in all cases and increased at higher potentials.

**Figure S11**. **Pair-pulsed electrical response of the PNCs.** (a) Current response of the PNCs under negative paired-pulse potentials. A pair of negative pulses was applied (-1 V, 0.5 s) with a small bias potential (0.05 V) to read the conductance. (b) Pair-pulsed depression index of the PNCs at varied pulse intervals. (c) Sequential pulsed-current responses measured at a low stimulation frequency of 0.1 Hz. (d) Enlarged baseline-current response during the low-frequency pulse-train measurement.

**Figure S12.** **Electrochemical impedance spectroscopy measurements of the PNCs.** (a) Experimental and fitting curves of the PNCs. The impedance spectrum shows a crossover point from the first quadrant to the fourth quadrant at a scanning frequency of about 20 mHz, corresponding to a memory time of about 50 seconds. The inset is the equivalent circuit for fitting the curve. EIS spectrum of the PNCs at varied (b) positive potentials or (c) negative potentials. All the cases exhibit a prominent inductive feature as shown in the fourth quadrant and enhanced with higher potential.

**Figure S13.** **Theoretical analysis of the current-time curves at electrical pulse stimulations.** Experimental and fitting current-time curves at (a) positive and (d) negative pulses. Simulated I-t curves predicted by the chemical inductor theory at (b) positive and (e) negative consecutive pulses. Calculated (c) PPF and (f) PPD index under consecutive pulse stimulations.

**Figure S14. Optical response of the PNCs.** (a) Current response of the PNCs at sequential pulse light illuminations, which reveals a notable optical memory. (b) Current-time curve of the PNCs under light illuminations (20 mW cm^-2^, 50 s). The PNCs show a long-term non-volatile current after light withdrawal.

**Figure S15.** **Wavelength dependence of the PNCs.** (a) UV-Vis spectrum of the PNCs. (b) I-T curves of the PNCs at different light wavelengths (100 mW cm^-2^) or in the absence of light illuminations.

**Figure S16. Working mechanism of light-enabled ionic transport in the PNCs.** (a) Schematic illustration of the ion transport mechanism through the PNCs under light illumination. Light exposure generates additional excess surface charge and creates an extra attractive electrostatic that facilitates cations entering the channel, thereby reducing the energy barrier for ion transport. (b) I-V curves of PNCs measured at different temperatures in 0.1 M KCl electrolytes. (c) Surface potential of the PNCs with and without light illuminations.

**Figure S17. Characteristics of the memristor-based FET.** (a) Schematic of the ionic FET. (b) Photo of the ionic FET. (c) Transfer (V_d_ = 0.05 V) and (d) output curves the ionic FETs measured in 1 mM KCl electrolyte. The gate electrolyte was LiPF6 solution. The inset of (c) is the enlarged leakage current of the ionic FETs measured in 1 mM KCl electrolyte. There is a slight leakage current for the ionic FET due to the intrinsic ion transport through the PNCs.

**Figure S18.** **Tunable output of the ionic FET.** (a) Transfer curves of the ionic FET under different light intensities measured in 1 mM KCl electrolyte (V_d_ = 0.05 V). (b) Current ratio of the ionic FET compared to the case without light illuminations (V_g_ = -1 V). (c) Transfer curves the ionic FET under different electrolyte concentration illuminated with 10 mW cm^-2^ (V_d_ = 0.05 V). (d) Current ratio at different electrolyte concentrations (V_g_ = -1 V).

**Figure S19.** Definition of relaxation time for a typical optoelectronic response under single electrical pulse, which is the duration for the inductive current change under electrical stimuli (ΔI) to recover to its 1/e.

**Figure S20.** **Datasets for pattern recognition.** The datasets contain handwritten numbers, sizes, or garments from MNIST, E-MNIST, and F-MNIST.

**Figure S21.** Recognition accuracies and losses during 1000 epochs for recognizing patterns from (a) E-MNIST and (b) F-MNIST datasets.

**Figure S22.** **Performance metrics for different recognition tasks.** The confusion matrix for (a) E-MNIST and (b) F-MNIST datasets.

**Figure S23.** Recognition accuracies and losses during 100 epochs for identifying moving particles in different directions.

**Figure S24.** **Tunable photoresponsivity of the ionic FET under different gate voltages (V_d_ = 0.05 V).** The photoresponsivity R ($R=\frac{\Delta I^{p}}{\Delta P}$) is extracted from Figure S18a, where Δ*I*^p^ and Δ*P* are the change of photocurrent and optical power, respectively. The photocurrent is calculated as *I*^p^= *I*-*I*^b^, i.e. subtracting dark current I^b^ from total output current I.

**Figure S25.** **Current-time curve of the ionic FET under light pulses (10 mW cm^-2^, 0.1 s).** At short pulse illuminations, the device can response in real-time.

**Figure S26. Implementation of the in-sensor computing.** (a) Experimental setup for implementing in-sensor computing. The setup integrates three key functionalities: (1) optical pattern input, where a programmable projector casts precise and dynamic light patterns onto the device array; (2) device gate control, where the gate of every OENFM device in the array is individually connected to external controllers and a computer, allowing for real-time and controllable modulation of the device's photoresponse via the applied gate voltage (V_g_); and (3) output current measurement, where the final calculated currents are measured using a high-precision source meter. (b) Electrical circuit of the OENFM array for in-sensor computing. The array is configured to consolidate the output signals into three independent current channels (I_1_, I_2_, I_3_). The connections strictly adhere to Kirchhoff's Law, realizing current summation at the output nodes. The photoresponse of each individual device is precisely set by the independent gate voltage (V_g_). This specific circuit configuration enables the array to distinguish distinct optical patterns, with the classification result determined by the relative current amplitude in the corresponding output channel.

**Figure S27.** Transfer curves the ionic FET array measured in 1 mM KCl electrolyte (V_d_ = 0.05 V). A_MN_ represents the Nth device in the Mth subpixel in the array.

**Figure S28.** (a) Transfer curves of an ionic FET array containing 27 devices. (b) Rectification ratio distribution of the ionic FET array.

**Figure S29.** **Simulated outputs for in-sensor optical pattern recognition.** (a) Accuracies and losses during 100 epochs for identifying optical patterns. (b) Simulated photocurrent for different optical patterns (‘H’, ‘K’, and ‘U’) illuminated on the device array. The clear separation of output current can be utilized for real pattern recognition.

**Table S1. Computing accuracy comparison with other physical reservoirs**

| **Device**  **structure** | **Type** | **Accuracy** | **Ref.** |
| --- | --- | --- | --- |
|  |  |  |  |
| Functionalized PEDOT:PSS channel | fluidic | 75.1% MNIST dataset | ^[7]^ |
| Tapered microfluidic channel | fluidic | 81% MNIST dataset | ^[8]^ |
| polyimide micropores | fluidic | 89.5% NNIST dataset | ^[9]^ |
| Nafion film | solid | 86.4% homemade wind discrimination dataset | ^[10]^ |
| optoelectronic polymers | solid | 88.18% MNIST dataset | ^[11]^ |
| Droplet interface synapses | fluidic | 86.2% MNIST dataset | ^[12]^ |
| **Photoresponsive nanofluidic channel** | **fluidic** | **91% MNIST dataset**  **97% homemade moving particle dataset**  **100% in-sensor computing** | **This work** |

**Table S2. Comparison with existing fluidic memristors**

| **Device structure** | **Mechanism** | **Synaptic plasticity** | **Functionality** | **Computing applications** | | | | **Ref.** |
| --- | --- | --- | --- | --- | --- | --- | --- | --- |
|  |  |  |  | **Logic gate** | **Classification** | **Prediction** | **Sensing** |  |
| Two-dimensional nanofluidic channels | Ionic self-assembly; surface adsorption. | STP; LTP; PPF; PPD; STDP | Electrical | / | / | / | / | ^[5]^ |
| Polyelectrolyte-confined fluidic channel | Surface charge effect | STP; LTP | Electrical | / | / | / | / | ^[13]^ |
| Highly asymmetric channels | Mechano–ionic effect | STDP | Electrical | Logic switch | / | / | / | ^[14]^ |
| MOF membrane | Surface charge effect | STP; LTP; PPF | Optical | / | / | / | / | ^[15]^ |
| Functionalized PEDOT:PSS channel | Photoelectrochemical effect | STP; SDP; HDP | Electrical Optical | / | MNIST dataset | / | / | ^[7]^ |
| Single-pore | Surface charge effect | PPD; PPF; SRDP | Electrical | Logic gate | / | / | / | ^[16]^ |
| Tapered microfluidic channel | Surface charge effect | STP | Electrical | / | MNIST dataset | / | / | ^[8]^ |
| **Photoresponsive nanofluidic channel** | **Surface adsorption** | **STP; STD; LTP; LTD; PPD; PPF** | **Electrical**  **Optical Optoelectrical** | **Logic gate** | **MNIST dataset** | **Moving particle** | **In-sensor computing** | **This work** |

# Reference

[1]	Y. Huang, H. Cheng, C. Yang, P. Zhang, Q. Liao, H. Yao, G. Shi, L. Qu, *Nat. Commun.* 2018, 9, 4166.

[2]	S. Tanaka, D. Nogami, N. Tsuda, Y. Miyake, *J. Colloid Interface Sci.* 2009, 334, 188.

[3]	J. Zheng, J. Chen, Y. Jin, Y. Wen, Y. Mu, C. Wu, Y. Wang, P. Tong, Z. Li, X. Hou, J. Tang, *Nature* 2023, 617, 499.

[4]	P. Robin, N. Kavokine, L. Bocquet, *Science* 2021, 373, 687.

[5]	P. Robin, T. Emmerich, A. Ismail, A. Niguès, Y. You, G.-H. Nam, A. Keerthi, A. Siria, A. K. Geim, B. Radha, L. Bocquet, *Science* 2023, 379, 161.

[6]	J. Bisquert, J. B. Roldan, E. Miranda, *Phys. Chem. Chem. Phys.* 2024, 26, 13804.

[7]	Z. Li, Q.-Q. Wu, Y. Wang, P. Lin, J.-J. Xu, H.-Y. Chen, W.-W. Zhao, *Adv. Mater.* 2026, 38, e07054.

[8]	T. M. Kamsma, J. Kim, K. Kim, W. Q. Boon, C. Spitoni, J. Park, R. van Roij, *Proc. Natl. Acad. Sci.* 2024, 121, e2320242121.

[9]	G. Guo, T. Xiong, B. Xie, J. Zhang, J. Zhang, Y. Lu, Y. Zhao, W. Ma, C. Pan, Y. Jiang, L. Mao, J. Wang, P. Yu, *Nat. Commun.* 2026, 17, 4275.

[10]	Z. Lv, H. Wang, J. Zheng, X. Zhao, G. Ding, Y. Wang, Y. Zhai, Q. Zhang, Y. Zhou, W. C. H. Choy, S.-T. Han, *Adv. Mater.* 2026, 38, e18105.

[11]	X. Wu, S. Wang, W. Huang, Y. Dong, Z. Wang, W. Huang, *Nat. Commun.* 2023, 14, 468.

[12]	Z. Li, S. K. Myers, J. Xiao, Y. Li, N. Noy, A. Leuski, A. Noy, *Sci. Adv.* 2025, 11, eadv6603.

[13]	T. Xiong, C. Li, X. He, B. Xie, J. Zong, Y. Jiang, W. Ma, F. Wu, J. Fei, P. Yu, L. Mao, *Science* 2023, 379, 156.

[14]	T. Emmerich, Y. Teng, N. Ronceray, E. Lopriore, R. Chiesa, A. Chernev, V. Artemov, M. Di Ventra, A. Kis, A. Radenovic, *Nat. Electron.* 2024, 7, 271.

[15]	J. Wang, Y. Jiang, T. Xiong, J. Lu, X. He, P. Yu, L. Mao, *Angew. Chem. Int. Ed.* 2025, 64, e202418949.

[16]	Y. Ling, L. Yu, Z. Guo, F. Bian, Y. Wang, X. Wang, Y. Hou, X. Hou, *J. Am. Chem. Soc.* 2024, 146, 14558.
